# A type-I diacylglycerol acyltransferase modulates triacylglycerol biosynthesis and fatty acid composition in the oleaginous microalga, *Nannochloropsis oceanica*

**DOI:** 10.1186/s13068-017-0858-1

**Published:** 2017-07-05

**Authors:** Hehong Wei, Ying Shi, Xiaonian Ma, Yufang Pan, Hanhua Hu, Yantao Li, Ming Luo, Henri Gerken, Jin Liu

**Affiliations:** 1 Institute for Food and Bioresource Engineering, Department of Energy and Resources Engineering and BIC-ESAT, College of Engineering, Peking University, Beijing, 100871 China; 2 Key Laboratory of Algal Biology, Institute of Hydrobiology, Chinese Academy of Sciences, Wuhan, 430072 China; 3 Institute of Marine and Environmental Technology, University of Maryland Center for Environmental Science and University of Maryland Baltimore County, Baltimore, MA 21202 USA; 4 Guangdong Provincial Key Laboratory of Applied Botany, Key Laboratory of South China Agricultural Plant Molecular Analysis and Genetic Improvement, South China Botanical Garden, Chinese Academy of Sciences, Guangzhou, 510650 China; 5 School of Sustainable Engineering and the Built Environment, Arizona State University Polytechnic campus, Mesa, AZ 85212 USA

**Keywords:** Diacylglycerol acyltransferase, Functional characterization, Genetic engineering, Microalga, *Nannochloropsis oceanica*, Triacylglycerol

## Abstract

**Background:**

Photosynthetic oleaginous microalgae are considered promising feedstocks for biofuels. The marine microalga, *Nannochloropsis oceanica,* has been attracting ever-increasing interest because of its fast growth, high triacylglycerol (TAG) content, and available genome sequence and genetic tools. Diacylglycerol acyltransferase (DGAT) catalyzes the last and committed step of TAG biosynthesis in the acyl-CoA-dependent pathway. Previous studies have identified 13 putative DGAT-encoding genes in the genome of *N. oceanica*, but the functional role of *DGAT* genes, especially type-I *DGAT* (*DGAT1*), remains ambiguous.

**Results:**

*Nannochloropsis oceanica* IMET1 possesses two *DGAT1* genes: *NoDGAT1A* and *NoDGAT1B*. Functional complementation demonstrated the capability of NoDGAT1A rather than NoDGAT1B to restore TAG synthesis in a TAG-deficient yeast strain. In vitro DGAT assays revealed that NoDGAT1A preferred saturated/monounsaturated acyl-CoAs and eukaryotic diacylglycerols (DAGs) for TAG synthesis, while NoDGAT1B had no detectable enzymatic activity. Assisted with green fluorescence protein (GFP) fusion, fluorescence microscopy analysis indicated the localization of NoDGAT1A in the chloroplast endoplasmic reticulum (cER) of *N. oceanica*. *NoDGAT1A* knockdown caused ~25% decline in TAG content upon nitrogen depletion, accompanied by the reduced C16:0, C18:0, and C18:1 in TAG *sn*-1/*sn*-3 positions and C18:1 in the TAG *sn*-2 position. *NoDGAT1A* overexpression, on the other hand, led to ~39% increase in TAG content upon nitrogen depletion, accompanied by the enhanced C16:0 and C18:1 in the TAG *sn*-1/*sn*-3 positions and C18:1 in the TAG *sn*-2 position. Interestingly, *NoDGAT1A* overexpression also promoted TAG accumulation (by ~2.4-fold) under nitrogen-replete conditions without compromising cell growth, and TAG yield of the overexpression line reached 0.49 g L^−1^ at the end of a 10-day batch culture, 47% greater than that of the control line.

**Conclusions:**

Taken together, our work demonstrates the functional role of NoDGAT1A and sheds light on the underlying mechanism for the biosynthesis of various TAG species in *N. oceanica.* NoDGAT1A resides likely in cER and prefers to transfer C16 and C18 saturated/monounsaturated fatty acids to eukaryotic DAGs for TAG assembly. This work also provides insights into the rational genetic engineering of microalgae by manipulating rate-limiting enzymes such as DGAT to modulate TAG biosynthesis and fatty acid composition for biofuel production.

**Electronic supplementary material:**

The online version of this article (doi:10.1186/s13068-017-0858-1) contains supplementary material, which is available to authorized users.

## Background

Triacylglycerols (TAGs) are energy-rich lipids widely used for human nutrition, chemical industries, and the production of high-energy density fuels [1]. Many oleaginous microalgae produce high levels of TAGs as the storage compounds [2–6], triggering substantial interest in exploring microalgae as biofuel feedstock. Efforts have been made for the further improvement of TAG yields, as TAG yields from the naturally occurring microalgal strains currently used for oil production are far less than the theoretical maximum [7, 8]. The understanding of pathways and regulatory mechanisms involved in lipid metabolism will help benefit the rational genetic manipulation of microalgae for improved oil production [9–11].

In higher plants, TAG biosynthesis has been documented and is believed to be mediated mainly via two pathways, acyl-CoA independent pathway and acyl-CoA-dependent Kennedy pathway [12]. The latter pathway starts from glycerol-3-phosphate with three sequential acylation steps, with the last step being mediated by a diacylglycerol acyltransferase (DGAT), which employs an acyl-CoA as the acyl donor and transfers the acyl moiety to the *sn*-3 position of DAG for TAG assembly [13]. DGAT catalyzes the only committed step in Kennedy pathway and is believed to play critical roles in TAG synthesis and accumulation. The well-characterized DGATs in higher plants include type-I (DGAT1) and type-II (DGAT2), the two structurally distinct groups of membrane-bound acyltransferases [14], and type-III (DGAT3), which is soluble and present in the cytosol [15]. Previous studies in higher plants have revealed distinct roles of different types of DGAT in TAG synthesis in different organisms [16, 17]. Bioinformatic analysis reveals the presence of *DGAT1* and *DGAT2* gene homologs in sequenced algal genomes [18–23]. Most of the current knowledge about the role of DGAT in algal TAG biosynthesis is derived from the model alga *Chlamydomonas reinhardtii*, in which DGAT2 has recently been characterized at the molecular and biochemical levels [24–26]. However, *Chlamydomonas* is generally not considered as an oleaginous organism for lipid production and may differ from oleaginous algae in lipid metabolism, driving the research interest to industrially important algae such as *Chlorella* and *Nannochloropsis* [21, 22, 27, 28].

*Nannochloropsis oceanica* has been recognized as an emerging model oleaginous alga in the study of TAG metabolism because of its fast growth, high TAG content, available genome sequence, and established genetic tools [20, 23, 29–32]. Thirteen putative DGAT-encoding genes were identified in the genomes of two *N. oceanica* strains: IMET1 [23] and CCMP1779 [20]. In the former strain, two out of the thirteen are annotated as *DGAT1* genes, *NoDGAT1A* and *NoDGAT1B*. RNA-Seq analyses following nitrogen depletion revealed the upregulation of *NoDGAT1A* and five *NoDGAT2*s [33], indicative of their roles in the TAG biosynthesis. In a recent study by Zienkiewicz et al. [28], one of the *DGAT2*s in *N. oceanica* was demonstrated to contribute to TAG biosynthesis. However, the role of *NoDGAT1*s in *N. oceanica* TAG synthesis remains ambiguous. Questions remain as to whether they are functional, how they are involved in TAG biosynthesis, and their potential in manipulating algae for improved lipid production.

By employing the industrially important oleaginous alga *N. oceanica*, we here functionally characterized algal *DGAT1* genes in depth by integrating the in silico, ex vivo, subcellular localization, in vitro and in vivo analyses. NoDGAT1A rather than NoDGAT1B shows acyltransferase activity. NoDGAT1A is likely localized in the chloroplast endoplasmic reticulum (cER), and prefers saturated/monounsaturated acyl-CoAs and eukaryotic DAGs for TAG biosynthesis. Overexpression experiments suggest the engineering potential of *NoDGAT1* in modulating TAG accumulation and fatty acid composition in microalgae. We discuss the role of NoDGAT1A in TAG metabolism and its implications for biotechnological applications in microalgae engineering.

## Results

### Growth, lipid variation, and *NoDGAT1* s expression upon nitrogen depletion

**Fig. 1:**
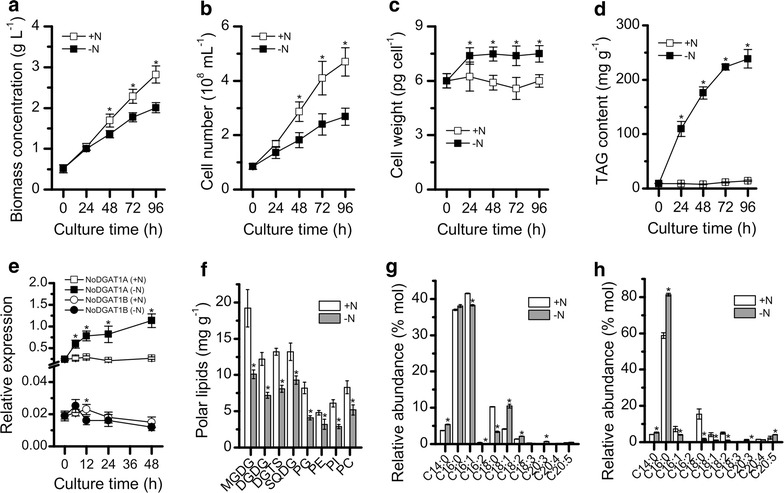
Growth and lipid profiles of *N. oceanica* in response to nitrogen depletion. **a**–**e** Time course of biomass concentration (**a**), cell number (**b**), cell weight (**c**), TAG content (**d**), and the transcriptional expressions of *NoDGAT1A* and *NoDGAT1B* (**e**). –N and +N represent nitrogen depletion and nitrogen repletion, respectively. **f** Polar lipid contents at 48 h of nitrogen depletion. **g**, **h** Relative abundance of fatty acids in TAG *sn*-1/3 (**g**) and *sn*-2 (**h**) positions at 48 h of nitrogen depletion. C18:1 represents the mixture of C18:1n9 and C18:1n7. Data are expressed as mean ± SD (*n* = 3).* Asterisks* indicate the significant difference compared with +N (*t*-test, P < 0.05)

Nitrogen is essential for algal growth and lipid metabolism. In response to nitrogen depletion, *N. oceanica* showed severely impaired growth, revealed by the considerably lower biomass concentration and cell number (Fig. 1a, b). The cell weight of nitrogen-depleted cells was higher than that of nitrogen-replete ones (Fig. 1c). Consistently, nitrogen-depleted cells accumulated substantial amounts of TAG, up to 230 mg g^−1^ cell dry weight, while nitrogen-replete cells synthesized only a basal level of TAG (<20 mg g^−1^) (Fig. 1d). DGAT is thought to play an important role in nitrogen-depletion-associated TAG accumulation in microalgae [26]. In *N. oceanica*, although a type-II *DGAT* gene has been recently characterized [28], the role of type-I *DGAT*s in TAG synthesis remains ambiguous. Here, two type-I *DGAT* genes, *NoDGAT1A* and *NoDGAT1B*, were examined at the transcriptional level. Obviously, the two genes were differentially regulated by nitrogen depletion: *NoDGAT1A* was upregulated moderately (up to fourfold increase), while *NoDGAT1B* had a much lower level of transcripts than *NoDGAT1A* and remained relatively stable in response to nitrogen depletion (Fig. 1e), suggesting that *NoDGAT1A* is more involved in the nitrogen-depletion-induced TAG biosynthesis. The induced TAG accumulation was accompanied by a decrease in membrane polar lipids including monogalactosyl diacylglycerol (MGDG), digalactosyl diacylglycerol (DGDG), diacylglycerol-N,N,N-trimethylhomoserine (DGTS), sulfoquinovosyldiacylglycerol (SQDG), phosphatidylglycerol (PG), phosphatidylethanolamine (PE), phosphatidylinositol (PI), and phosphatidylcholine (PC) (Fig. 1f), consistent with previous results in *Nannochloropsis* strains [30, 33] and indicative of the turnover of membrane lipids for TAG assembly under nitrogen-depleted conditions. Specifically, MGDG and DGDG, the major lipids of chloroplast, exhibited the most drastic reduction upon nitrogen depletion (Fig. 1f), consistent with the occurrence of shrunken chloroplast and reduced thylakoid membrane (Additional file 1: Figure S1).

The mass spectra of methylated fatty acids from TAG *sn*-1/*sn*-3 and *sn*-2 positions under different culture conditions are shown in Additional file 1: Figure S2. C16:0 and C16:1 represent the major fatty acids of TAG *sn*-1/*sn*-3 positions, which together account for over 75% of the fatty acids regardless of the culture conditions (Fig. 1g). Respective increase in C14:0, C18:1, and C18:2 was observed upon nitrogen depletion, with C18:1 having the largest increase, while C16:1 and C18:0 leveled off (Fig. 1g). In TAG *sn*-2 position, C16:0 is the predominant fatty acid, and it increased from 59% under nitrogen-replete conditions to 81% under nitrogen-depleted conditions, accompanied by decreases in C16:1, C18:0, C18:1, and C18:2 (Fig. 1h), suggesting that *N. oceanica* preferred to utilize the DAGs with *sn*-2 position being C16:0 for TAG synthesis upon nitrogen depletion.

### *NoDGAT1*s cloning and functional analysis in yeast

The cDNAs of *NoDGAT1A* and *NoDGAT1B* are 1314 and 2298 bp in length, encoding polypeptides of 427 and 765 amino acids, respectively. To gain insights into the evolutionary relationship between *NoDGAT1* s and other orthologs, a cladogram was reconstructed using MEGA6 [34] based on the multiple sequences from higher plants, animals, fungi, and algae. Phylogenetic analysis reveals that DGAT family proteins are clustered into three major groups: type-I, type-II and type-III (Additional file 1: Figure S3). NoDGAT1A and NoDGAT1B are closely related to the algal type-I DGAT orthologs. When analyzed on TMHMM Server 2.0 (http://www.cbs.dtu.dk/services/TMHMM/), NoDGAT1A and NoDGAT1B are predicted to have nine and eleven transmembrane domains, respectively (Additional file 1: Figure S4). Protein sequence alignment suggested that NoDGAT1s, NoDGAT1A in particular, contain conserved Motifs (Additional file 1: Figure S5), which are present in most type-I DGAT polypeptides [14].

**Fig. 2:**
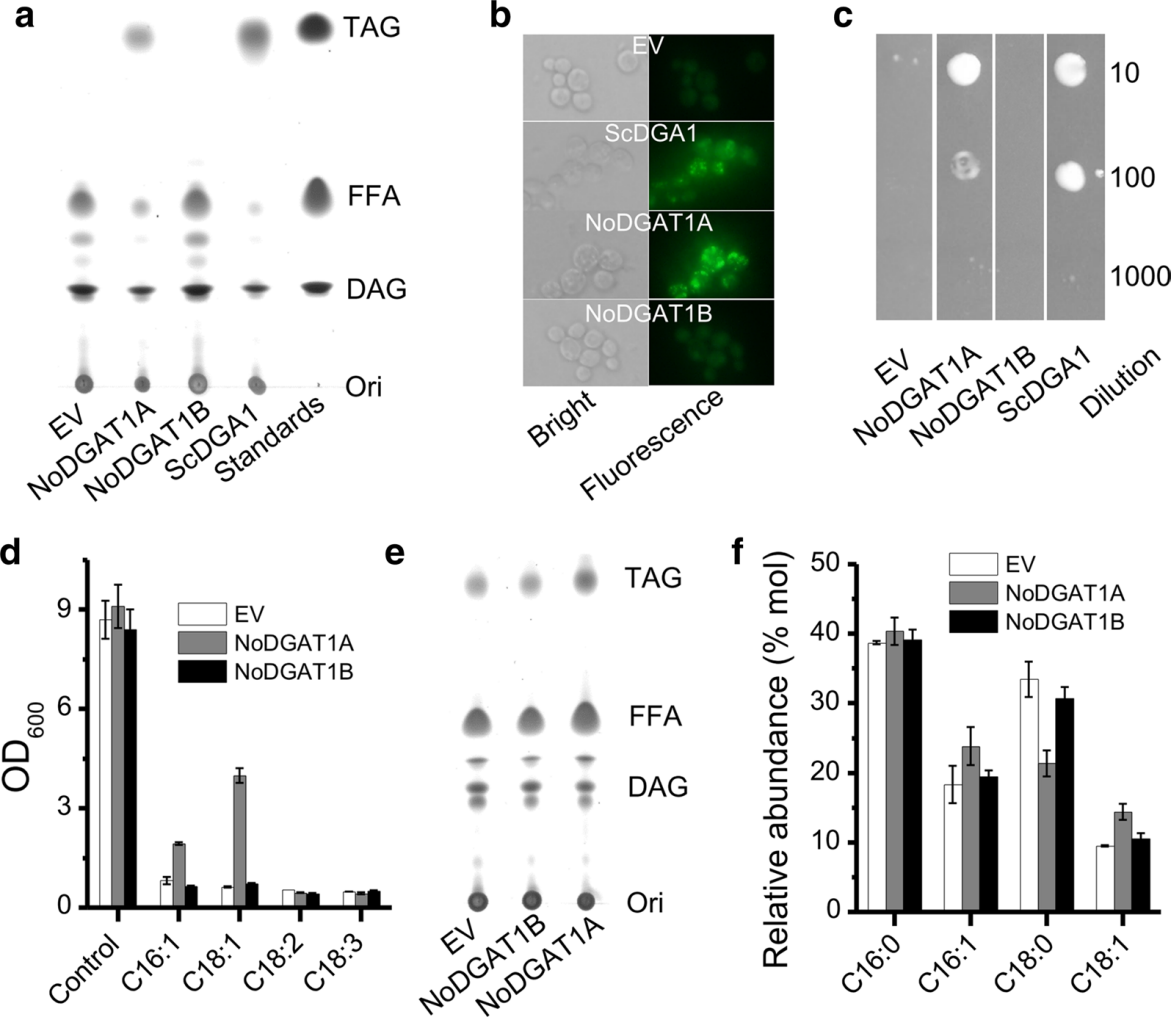
Functional characterization of *NoDGAT1*s in *Saccharomyces cerevisiae* cells. **a** TLC analysis of lipids extracted from TAG-deficient *S. cerevisiae* H1246 cells transformed with *NoDGAT1*s. Empty vector (EV) and ScDGA1 were used as the negative and positive controls, respectively. **b** BODIPY staining of *NoDGAT1*-expressing H1244 cells. *Left, bright field*; *right*, *fluorescent field*. *Green fluorescence* indicates the BODIPY-bound lipid droplets. **c** Growth of *NoDGAT1*-expressing H1244 cells (OD_600_, 1) in different dilutions (10- to 1000-fold) plated on 0.25 mM C18:1-containing solid medium for 3 days. 5 µL cells were plated. **d** Growth of *NoDGAT1*-expressing H1244 cells as affected by the feeding of free fatty acids of C16:1, C18:1, C18:2, and C18:3 after a 12-h cultivation. The concentration of FFAs was 125 µM. **e** TLC analysis of lipids extracted from *S. cerevisiae* INVSc1 cells transformed *NoDGAT1*s. **f** Relative abundance of fatty acids in TAG from *S. cerevisiae* INVSc1 cells transformed *NoDGAT1*s. Data in (**d**) and (**f**) are expressed as mean ± SD (*n* = 3)

To test the function of NoDGAT1 s, they were introduced into the TAG-deficient yeast strain H1246 for complementation. The transcriptional expression levels of the two genes were evaluated by quantitative real-time PCR (Additional file 1: Figure S6). The H1246 strain-overexpressing *ScDGA1*, a type-II DGAT from *Saccharomyces cerevisiae*, was used as the positive control. The expression of *NoDGAT1A* restored TAG biosynthesis in H1246 cells, as indicated by the prominent TAG spot on a TLC plate, which fractionated the lipid extracts from the transformed yeast cells (Fig. 2a), and the green fluorescence by BODIPY staining (Fig. 2b). By contrast, *NoDGAT1B* expression in H1246 cells failed to produce any detectable TAG (Fig. 2a, b), indicative of a nonfunctional encoded protein. Nevertheless, compared to *N. oceanica, S. cerevisiae* contains no such fatty acids as C14:0, C18:2, C20:4, and C20:5 (Fig. 1g, h), which may lead to the failure of functional complementation for *NoDGAT1B*.

The growth response of *DGAT*-expressing H1246 cells to free fatty acid feeding has been suggested as a feasible way for qualitative evaluation of the enzymatic activity and substrate specificity on acyl-CoAs [35, 36]. H1246 cells lack DGAT activity and may not efficiently metabolize the supplemented free fatty acids, leading to impaired cell growth; by contrast, heterologous expression of an active *DGAT* in H1246 cells enables sequestration of these fatty acids into TAG, and thus can restore cell growth to various extents depending on the enzymatic activity of DGAT. We first investigated the growth of H1246 cells carrying different vectors on agar plates containing 250 µM C18:1. As expected, the dotted H1246 cells carrying empty vector (EV, negative control) showed no growth on the plate, while the H1246 cells carrying *ScDGA1* grew and formed a white colony (Fig. 2c). Similar to the positive control, *NoDGAT1A*-carrying H1246 cells showed growth on the C18:1-containing plates. *NoDGAT1B*-carrying H1246 cells, on the other hand, did not survive, consistent with the functional complementation results (Fig. 2a). Next, four free fatty acids of C16:1, C18:1, C18:2, and C18:3n3 were used to test the growth of yeast cells in liquid cultures. The *NoDGAT1B*-carrying H1246 cells, similar to EV control, did not grow in the presence of any of the four fatty acids, further suggesting its encoded enzyme is nonfunctional toward these corresponding acyl-CoAs (Fig. 2d). By contrast, the *NoDGAT1A*-carrying H1246 cells exhibited differential growth responses: better growth with C18:1 than with C16:1 and no growth with C18:2 or C18:3 (Fig. 2d), indicating that NoDGAT1A has greater activity on monounsaturated acyl-CoAs than that on polyunsaturated ones.

The heterologous expressions of *NoDGAT1A* and *NoDGAT1B* in the TAG-producing *S. cerevisiae* strain INVSc1 were also investigated. The transcriptional expression levels of the two genes were evaluated with quantitative real-time PCR (Additional file 1: Figure S6).In comparison with the EV control, *NoDGAT1A*-carrying INVSc1 cells showed a considerable TAG increase, while *NoDGAT1B*-carrying INVSc1 cells produced TAG at levels similar to the EV control (Fig. 2e), consistent with the functional complementation data in H1246 cells (Fig. 2a). As for the TAG fatty acid profiles, the heterologous expression of *NoDGAT1A* rather than *NoDGAT1B* enabled an increase in the relative contents of both C16:1 and C18:1 at the expense of C18:0 (Fig. 2f).

### Substrate specificity of NoDGAT1A

**Fig. 3:**
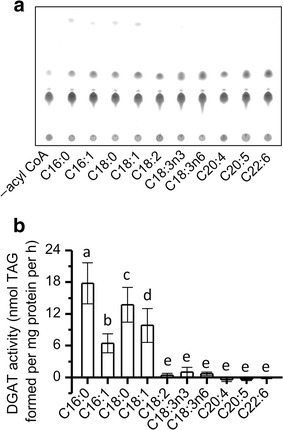
The in vitro substrate specificities of NoDGAT1A for acyl CoAs. **a** TLC analysis of lipids resulting from in vitro enzymatic reactions of NoDGAT1A with various acyl-CoAs. C18:1/C16:0-DAG was used as the acyl acceptor. **b** Quantification of the specific activity of NoDGAT1A for acyl-CoAs. Data in (**b**) are expressed as mean ± SD (*n* = 5), and those values designated by *different letters* are significantly different (P < 0.05)

The ability of NoDGAT1A to restore TAG biosynthesis in yeast led us to examine its substrate specificity. We have recently developed a nonradiolabeled DGAT in vitro assay [36], which allows for the measurement of activity and substrate specificity of DGAT and other acyltransferases toward a wide range of acyl-CoAs and DAGs with minimum background signals. Here, we first tested the preference of NoDGAT1A for acyl-CoAs (Fig. 3). Considering that the *sn*-2 position of TAG in *N. oceanica* consists mainly of 16-carbon acyls (Fig. 1h), the prokaryotic C18:1/C16:0-DAG was used as the acyl acceptor for the in vitro assay of NoDGAT1A toward ten various acyl-CoAs. NoDGAT1A preferred the saturated/monounsaturated acyl-CoAs over those polyunsaturated ones for the TAG synthesis (Fig. 3a). Quantitatively, NoDGAT1A had the highest activity on C16:0-CoA, followed by C18:0-, C18:1-, and C16:1-CoAs (Fig. 3b). When the acyl chain length was the same, NoDGAT1A preferred saturated acyl-CoAs; for instance, C16:0 and C18:0 led to a greater TAG level than C16:1 and C18:1, respectively (Fig. 3). These in vitro results suggested that NoDGAT1A contributes to the incorporation of more saturated fatty acids than that of the unsaturated ones into TAG in *N. oceanica*.

**Fig. 4:**
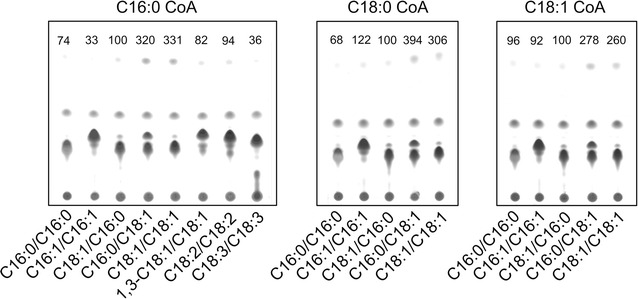
The in vitro substrate specificities of NoDGAT1A for DAGs. TLC analysis of lipids resulting from in vitro enzymatic reactions of NoDGAT1A with various DAGs. The acyl donor used in each reaction is indicated on the *panel*. The *numbers* above *each panel* indicate the relative TAG values, normalized to those obtained when C18:1/C16:0-DAG was used as the acyl acceptor (set as 100)

The specificity of NoDGAT1A for the other substrate DAG was also investigated (Fig. 4). Eight DAGs were tested, including seven 1,2-DAGs and one 1,3-DAG. Of the seven 1,2-DAGs, three were prokaryotic and four were eukaryotic. As NoDGAT1A showed the highest activity on C16:0-CoA in the above in vitro assay (Fig. 3), C16:0 CoA was first used as the acyl donor to test the DAG preference of NoDGAT1A. Apparently, NoDGAT1A preferred C16:0/C18:1- and C18:1/C18:1-DAGs over other DAGs tested (Fig. 4). When the acyl donor was C18:0- or C18:1-CoA, NoDGAT1A also demonstrated a strong preference for C16:0/C18:1- and C18:1/C18:1-DAGs over the other three tested prokaryotic DAGs. These data indicate that NoDGAT1A can utilize both prokaryotic and eukaryotic DAGs as the acyl acceptor for TAG formation, but prefers eukaryotic DAGs.

### Subcellular localization of NoDGAT1A

**Fig. 5:**
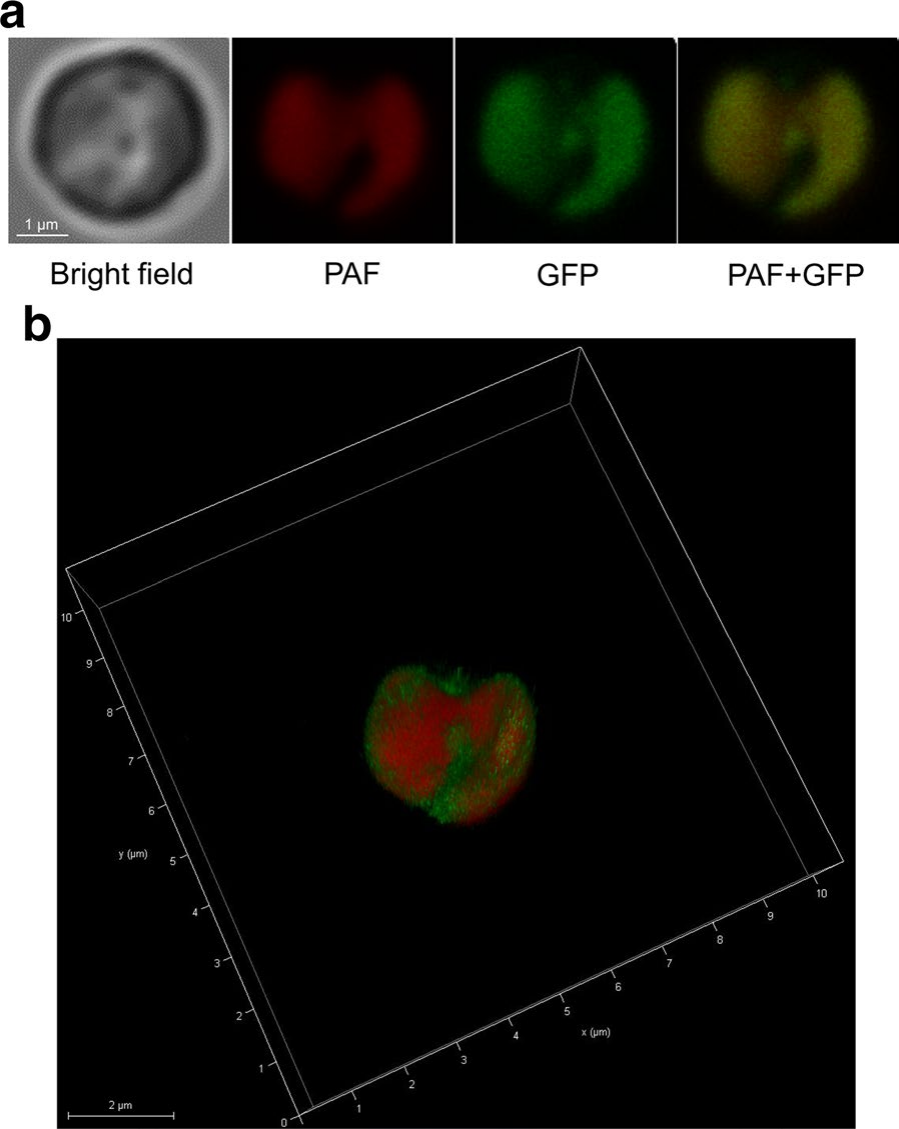
Subcellular localization of NoDGAT1A in *N. oceanica* cells. **a** Microscopy images of a representative transgenic line expressing NoDGAT1A-GFP fusion. From *right* to *left*: *bright field*, PAF (plastid autofluorescence), GFP, and merger of PAF and GFP (PAF + GFP). **b** 3D microscopy image of PAF + GFP

DGAT proteins are membrane bound and are thought to be ER-localized for TAG assembly in yeast and higher plants [37, 38]. The compartmentalization of DGAT in algae, however, has been rarely discussed earlier and remains ambiguous [26, 39]. We constructed the NoDGAT1A-GFP fusion and introduced it into *N. oceanica* for live cell observation, aiming to examine the subcellular localization of NoDGAT1A experimentally, which will aid in the understanding of its role in TAG biosynthesis. Clearly, the GFP signal was overlaid well with the plastid autofluorescence (PAF, Fig. 5a), indicative of its location in chloroplast rather than in cytosol, mitochondria, or ER [40]. As NoDGAT1A is a membrane-bound protein, it is unlikely to be positioned in stromal or intermembrane space of the chloroplast. To further clarify the compartmentalization, a 3D merger of GFP and PAF signals was built. Interestingly, GFP and PAF signals are not well matched, but instead GFP signal is clustered as an additional irregular layer surrounding PAF (Fig. 5b), suggesting that it is unlikely to be positioned in inner membrane envelope or outer membrane envelope. Considering that *Nannochloropsis* chloroplast evolved via secondary endosymbiosis and is delineated by four distinct membranes, with the outermost membrane being chloroplast ER (cER) connected to cytoplasmic ER [41], NoDGAT1A is probably localized in cER. Of course, the co-localization test with a protein known to localize to the cER would provide another layer of evidence for NoDGAT1A’s subcellular compartmentalization, which is worth investigating in future when such a marker protein is available for *N. oceanica*.

### *NoDGAT1A* knockdown impairs TAG synthesis and attenuates its relative content of saturated/monounsaturated fatty acids

**Fig. 6:**
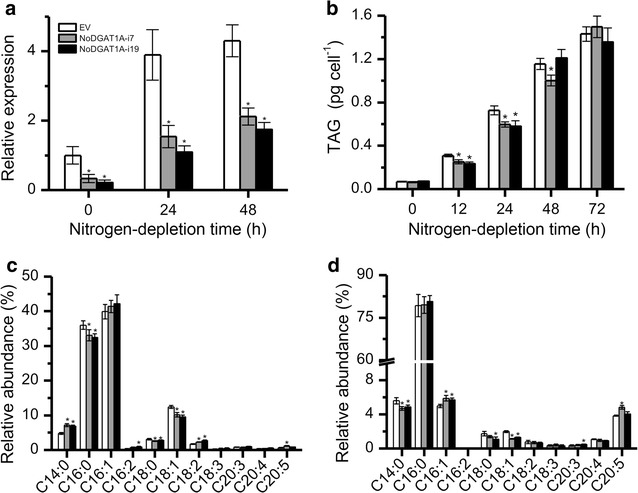
*NoDGAT1A* knockdown attenuated TAG accumulation and altered its fatty acid composition. **a** *NoDGAT1A* mRNA levels in *NoDGAT1A* knockdown lines and the empty vector (EV) control, as determined by quantitative real-time PCR. **b** TAG contents in *NoDGAT1A* knockdown lines and EV. **c**, **d** Relative abundance of fatty acids in the *sn*-1/*sn*-3 (**c**) and *sn*-2 (**d**) positions of TAG in *NoDGAT1A* knockdown lines and EV under nitrogen-depleted conditions (24 h). Data in (**a**–**d**) are expressed as mean ± SD (*n* = 3). Algal cells grown in nitrogen-replete medium for 4 days (considered as 0 h of nitrogen depletion) were used for nitrogen-depletion experiment. *Asterisks* indicate the significant difference compared with EV (*t*-test, P < 0.05)

In order to investigate the function and possible biological role of NoDGAT1A in *N. oceanica*, we first generated *NoDGAT1A* knockdown lines via an RNAi-mediated gene-silencing approach. Through screening over 30 putative transformants (confirmed by genomic PCR) by quantitative real-time PCR, two knockdown lines, NoDGAT1A-i7 and NoDGAT1A-i19, exhibited the maximum reduction in *NoDGAT1A* transcript (~80% lower than the control transformed with the empty vector) under both nitrogen-replete and nitrogen-depleted conditions (Fig. 6a).

To determine whether the knockdown of *NoDGAT1A* could influence TAG synthesis in *N. oceanica*, the TAG content per cell in the empty vector (EV) control and transformants were quantified. No difference in TAG content was observed between EV and *NoDGAT1A* knockdown lines under nitrogen-replete conditions (referred to 0 h of nitrogen depletion in Fig. 6b), although the *NoDGAT1A* expression in knockdown lines was markedly attenuated at the transcriptional level (Fig. 6a), indicating that NoDGAT1A is not involved in the nonstress-associated TAG biosynthesis. Upon stress induction, TAG contents in NoDGAT1A-i7 and NoDGAT1A-i19 decreased by 19 and 25%, respectively, compared with EV (12 h). As nitrogen-depletion stress persisted (24 h), the significant (*t* test, P < 0.05) TAG reduction in the knockdown lines remained prominent. However, no significant difference in TAG content was observed between EV and the knockdown lines after 48 h (Fig. 6b). When calculated as TAG content per dry weight or the ratio of fatty acids in TAG over total fatty acids (TFA), similar results of TAG reduction were observed for *NoDGAT1A* knockdown lines (Additional file 1: Figure S7).

To study the effect of *NoDGAT1A* knockdown on TAG fatty acid composition, we analyzed the fatty acids in TAG *sn*-1/*sn*-3 and *sn*-2 positions, respectively. Compared to EV, TAG showed a significant decline (*t*-test, P < 0.05) in the relative contents of C16:0, C18:0, and C18:1 in TAG *sn*-1/*sn*-3 positions of the knockdown lines under nitrogen-depleted conditions (24 h); by contrast, a significant increase (*t*-test, P < 0.05) occurred in the relative contents of C14:0 and C18:2 (Fig. 6c). The relative fatty acid contents in *sn*-2 position of TAG were also changed, with C14:0 and C18:1 being significantly (*t*-test, P < 0.05) decreased (Fig. 6d). These in vivo results from *NoDGAT1A* knockdown analysis were in agreement with the in vitro assay data that NoDGAT1A preferred the saturated and monounsaturated acyl-CoAs and eukaryotic DAGs (Figs. 3 and 4).

### *NoDGAT1A* overexpression promotes TAG synthesis and its relative content of saturated/monounsaturated fatty acids

**Fig. 7:**
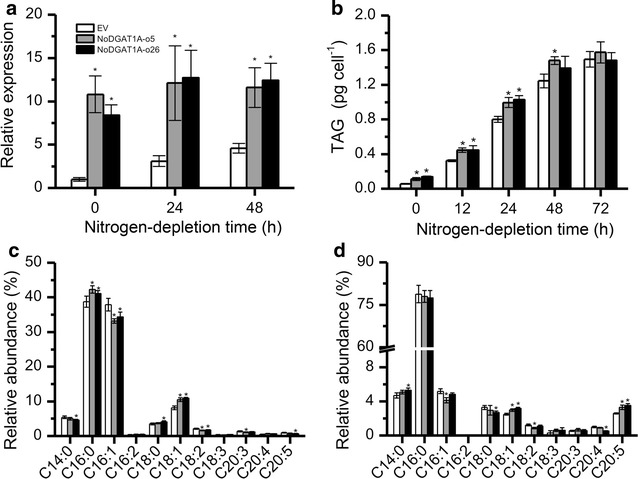
*NoDGAT1A* overexpression promoted TAG accumulation and altered its fatty acid composition. **a** *NoDGAT1A* mRNA levels in *NoDGAT1A* overexpression lines and EV, as determined by quantitative real-time PCR. **b** TAG contents in *NoDGAT1A* overexpression lines and EV. **c**, **d** Relative abundance of fatty acids in the *sn*-1/*sn*-3 (**c**) and *sn*-2 (**d**) positions of TAG in *NoDGAT1A* overexpression lines and EV under nitrogen-depleted conditions (24 h). Algal cells grown in nitrogen-replete medium for 4 days (considered as 0 h of nitrogen depletion) were used for nitrogen-depletion experiment. Data in (**a**–**d**) are expressed as mean ± SD (*n* = 3).* Asterisks* indicate the significant difference compared with EV (*t*-test, P < 0.05)

We also generated *NoDGAT1A* overexpression lines to investigate the functional role of NoDGAT1A in *N. oceanica*. Over fifty putative transformants (confirmed by genomic PCR) were screened by quantitative real-time PCR. Two overexpression lines, NoDGAT1A-o5 and NoDGAT1A-o26, which exhibited the maximal increases in *NoDGAT1A* transcripts (by ~10-fold higher than the control) were subsequently chosen for the analysis (Fig. 7a).

Unlike *NoDGAT1A* knockdown (Fig. 6b), *NoDGAT1A* overexpression led to considerable increases (1.9- and 2.4-folds for NoDGAT1A-o5 and NoDGAT1A-o26, respectively) in TAG contents per cell under nitrogen-replete conditions (referred to 0 h of nitrogen depletion in Fig. 7b). The TAG enhancement by *NoDGAT1A* overexpression was also observed at 12–48 h of nitrogen depletion (by 1.25- to 1.44-fold increase); but further nitrogen depletion to 72 h led to no difference (Fig. 7b). TAG enhancement caused by *NoDGAT1A* overexpression was also evidenced when calculated as TAG content per dry weight or the ratio of fatty acids in TAG over TFA (Additional file 1: Figure S8).

*NoDGAT1A* overexpression affected the fatty acid profiles in TAG *sn*-1/*sn*-3 and *sn*-2 positions (Fig. 7c, d). A significant increase (*t*-test, P < 0.05) was observed in the relative content of C16:0 and C18:1 in TAG *sn*-1/*sn*-3 positions, accompanied by a significant decrease (*t*-test, P < 0.05) in C16:1 and C18:2 (Fig. 7c). As for TAG *sn*-2 position, C18:1 and C20:5 showed a significant increase (*t*-test, P < 0.05) while C16:1 dropped significantly (*t*-test, P < 0.05) (Fig. 7d). In general, these *NoDGAT1A* overexpression results are in agreement with the in vitro assay data that NoDGAT1A preferred C16:0- and C18:1-CoAs and eukaryotic DAGs (Figs. 3 and 4).

### Heterologous expression of *NoDGAT1A* in *Chlamydomonas* enhances TAG accumulation

**Fig. 8:**
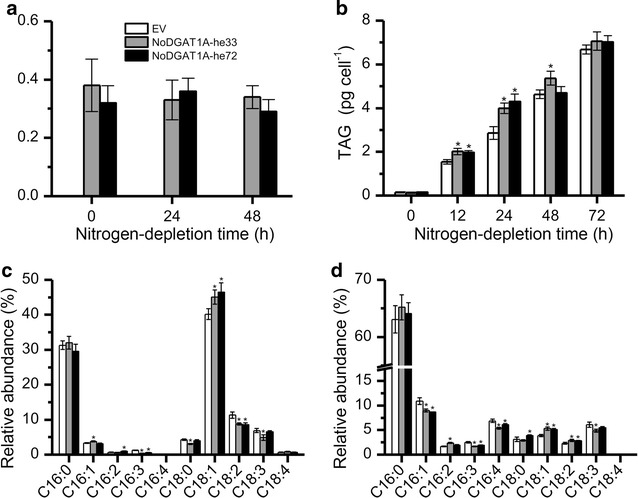
Heterologous expression of *NoDGAT1A* enhanced TAG synthesis in the *C. reinhardtii* strain UVM4. **a** *NoDGAT1A* mRNA levels (normalized to endogenous β-actin gene) in *NoDGAT1A* heterologous expression lines and EV, as determined by quantitative real-time PCR. **b** TAG contents in *NoDGAT1A* heterologous expression lines and EV. **c**, **d** Relative abundance of fatty acids in the *sn*-1/sn-3 (**c**) and *sn*-2 (**d**) positions of TAG in *NoDGAT1A* heterologous expression lines and EV under nitrogen-depleted conditions (24 h). Algal cells grown in nitrogen-replete medium for 4 days (considered as 0 h of nitrogen depletion) were used for nitrogen-depletion experiment. Data in (**a**–**d**) are expressed as mean ± SD (*n* = 3).* Asterisks* indicate the significant difference compared with EV (*t*-test, P < 0.05)

In order to see if *NoDGAT1A* has the potential to improve TAG synthesis in other algae, we introduced this gene into the model alga *Chlamydomonas reinhardtii*. The mutant strain UVM4, which shows improved transgene expression efficiency [42], was employed in the present study as a host for the heterologous expression of *NoDGAT1A*. More than one hundred putative transformants (confirmed by genomic PCR) were screened, and NoDGAT1A-he33 and NoDGAT1A-he72 showing the highest expression of *NoDGAT1A* (Fig. 8a) were selected for phenotype examination. Unlike in *N. oceanica*, *NoDGAT1A* expression in *C. reinhardtii* UVM4 had no effect on TAG accumulation under nitrogen-replete conditions (referred to 0 h of nitrogen depletion in Fig. 8b), possibly due to the difference in genetic traits of these two organisms (*N. oceanica* belongs to Eustigmatophyceae, while *C. reinhardtii* belongs to Chlorophyceae). By contrast, TAG enhancement was observed under nitrogen-depleted conditions, which is obvious at early stress stages (12–24 h) and TAG contents increased by 34 and 42% at 24 h for NoDGAT1A-he33 and NoDGAT1A-he72, respectively. However, the TAG increase became insignificant during the late stage of nitrogen depletion (48–72 h). The phenotype of TAG enhancement in heterologous expression lines of *Chlamydomonas* was also supported by the data of TAG content per dry weight or the ratio of fatty acids in TAG over TFA (Additional file 1: Figure S9).

The positional fatty acid profile of TAG from 24-h nitrogen-depleted algal samples was examined for both NoDGAT1A-he33 and EV control (Fig. 8c, d). *NoDGAT1A* heterologous expression led to significant increases (*t*-test, P < 0.05) in the relative contents of C16:1 and C18:1 in TAG *sn*-1/*sn*-3 positions, accompanied by the significantly (*t*-test, P < 0.05) attenuated C16:3 and C18:2 (Fig. 8c). As for TAG *sn*-2 position, C18:1 and C18:2 showed significant increases (*t*-test, P < 0.05) while C16:1, C16:3, and C16:4 exhibited significant decreases (*t*-test, P < 0.05) compared with EV control (Fig. 8d).

### Comparison of lipid productions between *NoDGAT1A* overexpression lines and EV control

**Fig. 9:**
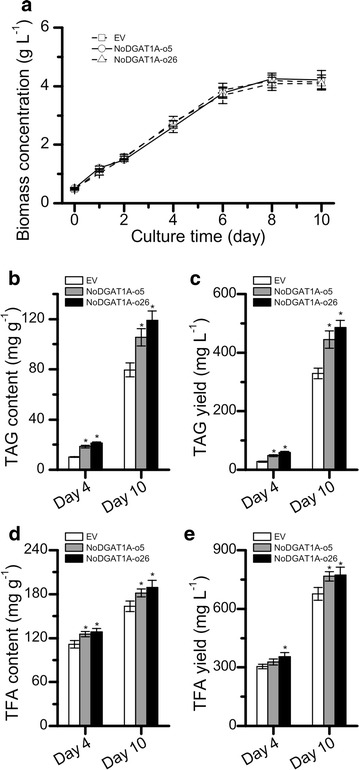
Comparison of growths (**a**), TAG contents (**b**), and yields (**c**), and TFA contents (**d**), and yield (**e**) between *NoDGAT1A* overexpression lines and EV control in a 10-day batch culture. The initial N concentration for the batch culture was 100 mg L^−1^. *Asterisks* indicate the significant difference compared to EV (*t*-test, P < 0.05)

Lipid production by microalgae depends not only on intracellular lipid content but also on biomass concentration. In order to evaluate the lipid-production performance of *NoDGAT1A* overexpression lines, a comparative analysis of biomass concentrations, TAG content and yield, and TFA content and yield was conducted for a 10-day batch culture under nitrogen-replete conditions. As indicated in Fig. 9a, no difference in biomass concentrations was observed between *NoDGAT1A* overexpression lines and EV control during the whole culture period. TAG content, on the other hand, showed a considerable difference: it reached 118.9 mg g^−1^ in the overexpression lines at the end of culture period, 50% higher than that in EV control (Fig. 9b). As regards the TAG yield, the overexpression lines reached 0.49 g L^−1^, 47% greater than that of EV control (Fig. 9c), indicative of the feasibility of the overexpressing *NoDGAT1A* for achieving improved TAG production. The overexpression of *NoDGAT1A* also led to a significant increase (*t*-test, P < 0.05) in the content (Fig. 9d) and yield (Fig. 9e) of TFA, although it was small compared with TAG increase.

## Discussion

### NoDGAT1A is functional and has distinctive substrate preference

Many microalgal strains are able to accumulate TAG in response to stress conditions, particularly nitrogen depletion [2]. The fatty acyls for TAG assembly can be either de novo synthesized or recycled from membrane polar lipids. In *N. oceanica*, the sharp increase in TAG was accompanied by an increase in TFA (Fig. 1d; Additional file 1: Figure S10), suggesting the contribution from the de novo fatty acid biosynthesis. This is further confirmed by the application of a specific fatty acid biosynthesis inhibitor, cerulenin, which led to a severe decrease in intracellular TAG content (Additional file 1: Figure S11). As TAG increases are accompanied by degradation of membrane lipids (Fig. 1f), the recycling of membrane lipids may be involved in TAG accumulation, which has been observed in several algal species [26, 43, 44]. It is generally agreed that glycerolipids with a 16-carbon acyl group in the *sn*-2 position are synthesized in plastid (prokaryotic pathway), while glycerolipids with an 18-carbon acyl group in *sn*-2 position are assembled at ER (eukaryotic pathway) in plants [13]. If this holds true in algae, *N. oceanica* may involve predominantly the prokaryotic pathway for TAG biosynthesis, as the *sn*-2 position of its TAG consists predominantly of 16-carbon acyl group (Fig. 1h).

DGAT catalyzes the committed and the last step in acyl-CoA-dependent TAG biosynthetic pathway and is critical for TAG accumulation in yeast and higher plants [9]. Compared with higher plants, microalgae possess a relatively high dose of annotated *DGAT* genes (5–13; [20, 21, 23, 45, 46]). The presence of high doses of *DGAT* genes suggests distinct yet complicated TAG biosynthesis and regulation mechanisms in *N. oceanica*. In accordance with higher TAG content upon nitrogen depletion, *NoDGAT1A* showed a great increase at the mRNA level, while *NoDGAT1B* had almost no change (Fig. 1d, e), indicating that *NoDGAT1A* contributes to stress-associated TAG accumulation. Functional complementation also indicated that the expression of *NoDGAT1A* but not *NoDGAT1B* restored TAG accumulation in H1246 cells (Fig. 2a). Even when the cells were fed with certain fatty acids (e.g., C18:2, C18:3, 20:4, 20:5) that are present in *N. oceanica* but not in yeast cells, no detectable TAG was observed for *NoDGAT1B*-carrying H1246 cells (Additional file 1: Figure S12). Consistent with the ex vivo data in H1246 (Fig. 2), in vitro DGAT assay demonstrated the enzymatic activity of NoDGAT1A (Fig. 3) rather than NoDGAT1B (Additional file 1: Figure S13). Of course, we cannot exclude the possibility that the functional failure of *NoDGAT1B* may be due to the codon usage preference of yeast. It is also possible that the predicted NoDGAT1B might not be a real DGAT but in fact a monoacylglycerol acyltransferase or other type of acyltransferase, since this distinction is based only on sequence data.

We have recently developed a nonradiolabeled in vitro assay for qualification and quantification of DGAT activity, which is featured by the use of widely available and regular acyl-CoAs and DAGs [36]. By means of this assay, a wide range of acyl-CoAs present in *N. oceanica* were evaluated with NoDGAT1A, revealing its preference for saturated/monounsaturated acyl-CoAs (Fig. 3). Notably, NoDGAT1A showed little in vitro activity on C20:5, although it is relatively abundant in *N. oceanica* [47], which may partially explain the extremely low abundance of C20:5 in TAG (Additional file 1: Figure S14). Interestingly, *C. reinhardtii* is devoid of C20:5, but its DGATs such as CrDGTT1 and CrDGTT2 showed respective high activity on C20:5-CoA in an in vitro assay [26], indicating that these DGATs may have potential in the engineering of *N. oceanica* for C20:5-enriched TAG production. DAG is the other substrate of DGAT, but the preference of DGAT on DAG has been less studied, and only a limited number of DAG species have been evaluated [17, 48]. In the present study, we evaluated the preference of NoDGAT1A for a wide range of DAGs in vitro. Interestingly, NoDGAT1A showed a strong preference for eukaryotic C16:0/C18:1- and C18:1/C18:1-DAGs over prokaryotic (C16:0/C16:0, C16:1/C16:1, and C18:1/C16:0) DAGs (Fig. 4), indicative of its greater contribution to eukaryotic TAG accumulation. Similarly, we have recently reported a type-II DGAT from *C. reinhardtii* (CrDGTT2) with a preference for eukaryotic DAGs [26]. Although *DGAT1* from higher plants has been well characterized [16, 17, 49–51], there are several reports mostly regarding *DGAT1* from algae including *Phaeodactylum tricornutum* [52], *Myrmecia incisa* [53], *Chlorella vulgaris* [54], and *Chlorella ellipsoidea* [55]. However, these studies about algal *DGAT1* gene characterization were mainly restricted to functional complementation in TAG-deficient yeast strains, without investigating the in vitro enzymatic activity or substrate preference. This work to our knowledge represents the first report of characterizing the in vitro activity and substrate preference of algal DGAT1 covering a wide range of acyl-CoAs and DAGs.

### NoDGAT1A is critical for stress-associated TAG biosynthesis in *N. oceanica*

There were no in vivo experiments for evaluating algal *DGAT1* function in previous studies [52–55]. In the present study, we employed the RNAi-mediated gene silencing and overexpression methods to generate *NoDGTAT1A*-knockdown and-overexpression lines, respectively, for in vivo functional analysis. It is not surprising that *NoDGAT1A* knockdown had no effect on TAG level under nitrogen-replete conditions, as phospholipid:DAG acyltransferases instead of DGAT are thought to be the major contributors to TAG synthesis under nonstress conditions in algae [56]. The significant decrease (Fig. 6b, 12–24 h) in TAG level caused by *NoDGAT1A* knockdown suggested its important role in nitrogen-depletion-associated TAG synthesis in *N. oceanica*. *NoDGAT1A* overexpression, on the other hand, enabled *N. oceanica* to synthesize more TAG (Fig. 7b), further confirming its in vivo function in *N. oceanica*. It is worth noting that the TAG content was not significantly altered at the late stage of nitrogen-depleted conditions for both *NoDGAT1A* knockdown (Fig. 6b, 48 h and thereafter) and *NoDGAT1A* overexpression (after 48 h), possibly due in part to the decreased *NoDGAT1A* knockdown efficiency (Fig. 6a) and overexpression level (Fig. 7a), respectively. In addition, many other acyltransferases in *N. oceanica* showed upregulation upon nitrogen depletion [33], which may be functional and compromise the phenotype caused by *NoDGAT1A* knockdown or overexpression. Interestingly, *NoDGAT1A* overexpression also led to a remarkable TAG increase under nitrogen-replete conditions (Fig. 7b). Consistent with the enhanced TAG levels, *NoDGAT1A* transcripts exhibited a drastic increase (by ~10-fold) in the overexpression lines under nitrogen-replete conditions (Fig. 7a), even higher than that in the wild type under nitrogen-depleted conditions (Fig. 1e). This may partially explain that *NoDGAT1A* overexpression enabled *N. oceanica* to synthesize more TAG, while its knockdown had no effect on TAG level under nitrogen-replete conditions (Figs. 6b, 7b). It has been recently reported that in *C. reinhardtii,* TAG attenuation caused by *DGAT* knockdown was accompanied by changes in membrane lipids [26]. In the present study, we did not notice a similar effect of *NoDGAT1A* knockdown on *N. oceanica* membrane lipids (Additional file 1: Figure S15).

As DGAT contributes primarily to the acyl groups in the *sn*-3 position of TAG, the TAG positional analysis may be more informative. The fatty acids in *sn*-1 and *sn*-3 positions cannot be distinguished from each other by the currently available methods and are normally grouped as a fraction separated from *sn*-2 fatty acids by the action of *Rhizopus arrhizus* lipase [26, 57, 58]. Our TAG positional analysis indicated that *NoDGAT1A* knockdown and overexpression each led to differential changes in the fatty acid profiles of TAG *sn*-1/*sn*-3 and *sn*-2 positions (Figs. 6c, d, 7c, d), consistent with the in vitro assay results that NoDGAT1A had distinct substrate specificity on acyl-CoAs and DAGs (Figs. 2 and 3). By combining the ex vivo, in vitro, and in vivo data, we inferred that NoDGAT1A is likely to contribute to transferring saturated and monounsaturated acyl groups (C16:0, C16:1, C18:0, and C18:1) to eukaryotic DAGs for TAG synthesis via the Kennedy pathway.

In *C. reinhardtii*, TAG precursors are thought to be synthesized mainly from the prokaryotic pathway in chloroplast [26, 57, 59, 60]. Similarly, *N. oceanica* TAG contained predominantly C16 acyl groups in its *sn*-2 position (Fig. 1h), suggesting a role of prokaryotic pathway for TAG synthesis. Our in vitro and in vivo data revealed that NoDGAT1A accepted both prokaryotic and eukaryotic DAGs for TAG assembly; however, NoDGAT1A had a strong preference for eukaryotic DAGs, C16:0/C18:1, and C18:1/C18:1 in particular (Fig. 3), suggesting its role in eukaryotic TAG biosynthesis. Thus, NoDGAT2s or other unknown acyltransferases may contribute more to prokaryotic TAG biosynthesis, which needs to be further validated experimentally. NoDGAT1A harbors the sequence YYH/AD (Additional file 1: Figure S4), a putative endoplasmic reticulum (ER) retrieval motif [17], indicative of its localization to the ER. To unravel the subcellular localization of NoDGAT1A experimentally, we constructed NoDGAT1A-GFP fusion, and the results indicate that NoDGAT1A is likely to reside in the cER (Fig. 5). cER bridges the chloroplast and ER and may serve as the transiting sites for lipid trafficking between these two organelles, similar to the chloroplast–ER membrane contact sites in higher plants [61]. The subcellular localization of NoDGAT1A in cER may facilitate its access to the substrates of DAGs and acyl-CoAs from both organelles.

**Fig. 10:**
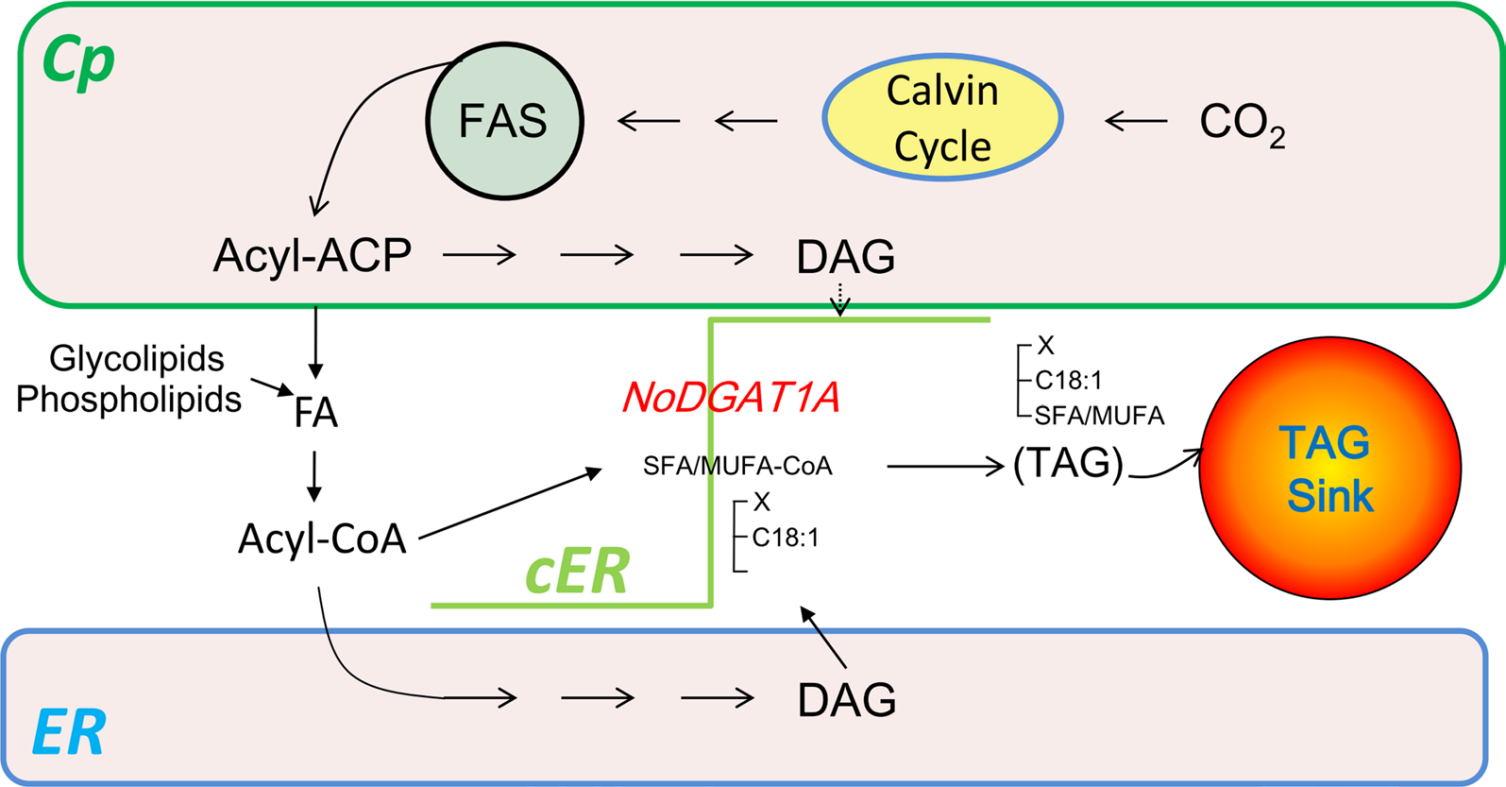
A hypothesized working model illustrating the role of NoDGAT1A in TAG biosynthesis in *N. oceanica.* For simplicity, a single lipid droplet (TAG sink) is shown bridging the endoplasmic reticulum (ER) and chloroplast (Cp) envelop. The de novo-synthesized fatty acids, together with those released from glycolipids and phospholipids, constitute the acyl-CoA pool. NoDGAT1A, probably located in cER, utilizes eukaryotic DAGs and saturated/monounsaturated (SFA/MUFA)-CoAs for TAG assembly. Not all intermediates or reactions were displayed. For simplicity, cER is expressed as an elbow-like line linking ER and chloroplast envelope. *ER* endoplasmic reticulum, *Cp* chloroplast, and *cER* chloroplast ER

Taking all these results together, we proposed a working model for the role of NoDGAT1A in TAG biosynthesis in the oleaginous alga *N. oceanica* (Fig. 10). The fatty acyls de novo synthesized and/or recycled from the turnover of membrane lipids constitute the acyl-CoA pool and enter Kennedy pathway for the synthesis of both prokaryotic and eukaryotic DAGs. NoDGAT1A, which is upregulated upon nitrogen depletion, accumulates and resides in cER and accesses mainly eukaryotic DAGs and C16–C18 saturated/monounsaturated acyl-CoAs to synthesize eukaryotic TAG species packed into lipid droplets (LDs, the TAG sink).

### Biotechnological implications

DGAT has gained increasing interest in various organisms including algae, and its engineering potential for oil production in yeast and higher plants has been well documented [62–66]. We here conducted the in-depth characterization of *NoDGAT1A* from *N. oceanica*, an oleaginous and industrially important marine alga. Both the overexpression of *NoDGAT1A* in *N. oceanica* and the heterologous expression of *NoDGAT1A* in the green alga *C. reinhardtii* enhanced the TAG biosynthesis and the relative content of C18:1 in TAG (Figs. 7 and 8), indicative of its potential application in algae engineering for improved oil production. It has been suggested that C18:1 helps benefit the balance between oxidative stability and low-temperature properties and promote the quality of biodiesel [67]. In this context, DGATs with preference for C18:1 such as NoDGAT1A are preferred for genetic engineering of algae for biodiesel production applications. *NoDGAT1A* overexpression in *N. oceanica* promoted TAG synthesis not only under nitrogen-depleted but also under nitrogen-replete conditions without compromising algal growth leading to considerably higher TAG yield (Figs. 7b and 9a). This is particularly important as it may allow using nonstressed algal cells instead of the stressed ones (typically associated with impaired growth and thus compromised oil production) for oil production. Although efforts to overexpress *NoDGAT2* have been made to enhance TAG synthesis in *N. oceanica*, the significance is partly compromised by reduced algal growth [28, 68]. In this context, *NoDGAT1A* may be superior to *NoDGAT2*s in genetic engineering of oleaginous algae for improved TAG synthesis and production. The difference in algal growth caused by overexpression of *NoDGAT1A* and *NoDGAT2*s may be due to the different properties of these two types of *DGAT* gene. It may also result from the use of different expression vectors with different promoters.

Bioinformatic analysis reveals the presence of multiple conserved motifs in NoDGAT1A ([14]; Additional file 1: Figure S4), which may play an important role in the acyltransferase activity across the DGAT1 family. Previous studies revealed that amino acid mutations occurring in these motifs led to considerably enhanced acyltransferase activity of DGAT1 from *Tropaeolum majus* [50] and *Zea mays* [69]. These findings point to the possible manipulation of *NoDGAT1A* for improved enzymatic activity and bioengineering uses in oil production. In addition, stronger promoters, which have high expression levels under nitrogen depletion such as *LDSP* promoter [20], are needed to drive *DGAT* genes with an aim to maintain TAG enhancement during the late stage of nitrogen-depleted conditions. Future genetic approaches for the further improvement of algal oils may lie in the engineering of multiple genes, e.g., overexpression of *DGAT* (pulling carbon flux toward TAG) together with transcription factors ([63, 65]; upregulating fatty acid/TAG biosynthetic pathway globally to push carbon flux toward TAG) and/or downregulation of TAG lipase genes ([70]; and protecting TAG from degradation).

## Conclusions

Several studies have reported the characterization of algal type-I *DGAT* genes, but have been restricted to sequence analysis, functional complementation in yeast and transcriptional expression levels in algae [52–54], and heterologous expression in higher plants [55]. Here, we have solidly demonstrated the function and critical role of *NoDGAT1A* for TAG biosynthesis in the oleaginous alga *N. oceanica* by functional complementation in the TAG-deficient *S. cerevisiae* strain H1246 (Fig. 2), in vitro DGAT assay for substrate specificity (Figs. 3 and 4), knockdown (Fig. 6), and overexpression (Fig. 7). *NoDGAT1A* is likely positioned in cER (Fig. 5), where it prefers to access and utilize saturated/monounsaturated acyl-CoAs and eukaryotic DAGs for TAG biosynthesis. From a biotechnological point of view, *NoDGAT1A* is a useful engineering target for promoting cellular TAG accumulation, which has been evidenced in yeast (Fig. 2), the modal alga *Chlamydomonas* (Fig. 8) and the oleaginous alga *N. oceanica* (Fig. 7). Robust algal TAG production is hampered by the typically required nutrient stress, which is associated with severely compromised biomass production. Unlike *NoDGAT2*s [28, 68], *NoDGAT1A* overexpression in *N. oceanica* enhanced TAG synthesis considerably but without slowing down biomass production under nitrogen-replete conditions (Fig. 9), indicating its potential in industrial uses. More importantly, *NoDGAT1A* expression increased the abundance of C18:1 in TAG (Figs. 7 and 8), which is beneficial to algal lipid-derived biodiesel as C18:1 helps enhance the quality of biodiesel [67]. These results not only shed light on the underlying mechanism for the biosynthesis of various TAG species in *N. oceanica,* but also provide insights into genetic engineering of microalgae by manipulating rate-limiting enzymes such as DGAT to modulate TAG synthesis and fatty acid composition for biofuel production.

## Methods

### *Nannochloropsis* strain and growth conditions

The marine microalga *Nannochloropsis oceanica* IMET1 was from the Institute of Marine and Environmental Technology, the University Systems of Maryland. It was maintained at 16 °C on an agar plate of the modified F/2 medium (100 mg L^−1^ N and 4.5 mg L^−1^ P) containing 20 g L^−1^ sea salt [71]. In brief, 10 mL of modified F/2 liquid medium was inoculated with cells from plates, and the alga was grown aerobically in flasks at 25 °C for 6 days (hand shaking twice per day) illuminated with continuous light of 30 µE m^−2^ s^−1^. The algal cells were then inoculated at 10% (v/v) into 250-mL columns (3-cm diameter) provided with constant illumination of 70 µE m^−2^ s^−1^ and aeration of 1.5% CO_2_ enriched air. Cells grown with nitrogen for 4 days (linear growth phase) were collected, washed three times with nitrogen-free medium, and resuspended in nitrogen-free medium for nitrogen-depletion experiments. For the inhibition of fatty acid biosynthesis, cerulenin (Sigma-Aldrich, St. Louis, MO, USA), which specifically inhibits β-ketoacyl-ACP synthase I (KAS I), was added upon N-depletion at a concentration of 10 µM, according to Liu et al. [72].

### Sequence analysis

TargetP 1.1 (http://www.cbs.dtu.dk/services/TargetP/ ) and SignalP 4.1 (http://www.cbs.dtu.dk/services/SignalP/) were used for the prediction of transit peptides of NoDGAT1A and NoDGAT1B. Sequence alignment of DGAT polypeptides was conducted using ClustalX2.1 (http://www.clustal.org/clustal2/), and the phylogenetic tree was generated using MEGA6 [34]. Proteins used for analysis are listed in Additional file 2: Table S1.

### RNA isolation and quantitative real-time PCR

RNA was isolated using the TRI Reagent (Invitrogen, Carlsbad, CA, USA) according to the manufacturer’s instructions. The total RNA concentration was determined using NannoDrop 2000c (Thermo Scientific, Wilmington, Delaware, USA) and the quality was checked by electrophoresis. The cDNA synthesis and quantitative real-time PCR were performed as described by Liu et al. [72] using a 7500 Fast Real-Time PCR System (Applied Biosystems, Waltham, MA, USA) with SYBR Green PCR Master Mix (Invitrogen). Primer sequences used for quantitative real-time PCR are listed in Additional file 2: Table S2. The mRNA expression level was normalized using the actin gene as the internal control.

### *NoDGAT1* gene cloning and expression in yeast

The type-I diacylglycerol acyltransferase genes from *N. oceanica* including *NoDGAT1A* (GenBank Accession No. KY073295) and *NoDGAT1B* (GenBank Accession No. KY073296) were PCR amplified using cDNA as template and cloned into the yeast expression vector pYES2-CT (Invitrogen). PCR primers for cloning are listed in Additional file 2: Table S2. After confirmation by restriction enzyme digestion and sequencing, the recombinant pYES2-NoDGAT plasmids were transformed into the *S. cerevisiae* TAG-producing strain INVSc1 or TAG-deficient quadruple mutant strain H1246 by means of S.c. EasyComp Transformation Kit (Invitrogen). Colony PCR and quantitative real-time PCR were used to verify the presence of the plasmids and the expression of *DGAT1*s, respectively, in the transformants. H1246 cells carrying the empty vector pYES2-CT (EV control) and pYES2-ScDGA1 (containing a type-2 *DGAT* gene from S. *cerevisiae*) were from Liu et al. [26] and Liu et al. [36], respectively. *NoDGAT1*s expression induction by galactose was performed as previously described [36]. When necessary, fatty acids were fed to yeast cultures as described by Siloto et al. [35], with supplementation of (palmitoleic acid) C16:1, oleic acid (C18:1), linoleic acid (C18:2), and α-linolenic acid (C18:3n3) at a concentration of 125 µM upon galactose induction.

### Yeast microsome preparation and in vitro DGAT activity assay

The H1244 transformants bearing *NoDGAT1* s were grown in SC-uracil medium with 2% galactose for 12 h at 30 °C. Cells were then harvested and washed twice with ice-cold distilled water. The cell pellets were resuspended in cell lysis buffer (containing 5% glycerol, 20 mM Tris–HCl (pH 8.0), 0.3 M ammonium sulfate, 10 mM MgCl_2_, 1 mM EDTA, 1 mM DTT, 1 × EDTA-free Protease Inhibitor Cocktail Set X (Calbiochem), 1 mM PMSF) to an OD_600_ of approximately 100 and lysed by passing twice through a French pressure cell (Spectronics Instruments, Rochester, NY, USA) at an internal pressure of 15,000 PSI. Cell debris was removed from the suspension by centrifugation at 10,000×*g* for 10 min at 4 °C, and the supernatant was centrifuged further at 100,000×*g* for 2 h at 4 °C. The resulting microsomal membrane pellets were resuspended in microsome storage buffer (50 mM Tris–HCl, pH 7.5, 10% glycerol) to yield a protein concentration of 10 µg µL^−1^ for immediate use or later use, storing at −80 °C.

The in vitro DGAT activity assay was conducted according to our previously described procedures [26, 36]. The acyl-CoAs tested included palmitoyl-CoA (C16:0-CoA), hexadecenoyl-CoA (C16:1-CoA), stearoyl-CoA (C18:0-CoA), oleoyl-CoA (C18:1-CoA), linoleoyl-CoA (C18:2-CoA), α-linolenoyl-CoA (C18:3n3-CoA), γ-linolenoyl-CoA (C18:3n6-CoA), eicosapentaenoyl-CoA (C20:5-CoA), and docosahexaenoyl-CoA (C22:6-CoA). The DAGs tested were C16:0/C16:0-, C16:1/C16:1-, C18:1/C16:0-, C16:0/C18:1-, C18:1/C18:1-, 1,3-C18:1/C18:1-, C18:2/C18:2-, and C18:3/C18:3-DAGs. C16:1/C16:-, C18:2/C18:2-, and C18:3/C18:3-DAGs were purchased from Larodan Fine Chemicals (Malmo, Sweden), whereas C18:1/C16:0-DAG was prepared by partial digestion of C18:1/C16:0/C18:1-TAG (Larodan Fine Chemicals) with *Rhizopus arrhizus* lipase (Sigma-Aldrich) and recovery of DAG. All other lipid standards were purchased from Avanti Polar Lipids (Alabaster, AL, USA).

### Constructs for subcellular localization, knockdown, and overexpression of *NoDGAT1A* in *N. oceanica* and nuclear transformation

For the subcellular localization vector, the coding sequence of *NoDGAT1A* was fused upstream of GFP under the control of violaxanthin/chlorophyll a-binding protein 2 (VCP2) promoter (Additional file 1: Figure S16a), as described by Moog et al. [40]. The RNAi vector construction for *NoDGAT1A* knockdown followed the procedures described by Wei et al. [32], and the resulting vector is depicted in Additional file 1: Figure S16b. For the overexpression vector, *NoDGAT1A* coding sequence was driven by the *Nannochloropsis* ubiquitin extension protein promoter (Additional file 1: Figure S16c). Nuclear transformation of *N. oceanica* was performed by electroporation according to Li et al. [29]. Transformants were selected on modified F/2 plates with 2.5 μg mL^−1^ zeocin (Life Technologies, Carlsbad, CA, USA) and verified by genomic PCR. Quantitative real-time PCR was employed to determine the knockdown efficiency and overexpression of *NoDGAT1A*. The live-cell fluorescence observation for NoDGAT1A-GFP fusion transgenic lines was performed using a Leica TCS SP8 laser scanning confocal microscope (Germany). Fluorescence of GFP and chlorophyll autofluorescence was exited at 488 nm with the emission detected at a bandwidth of 500–525 and 650–750 nm, respectively.

### Heterologous expression of NoDGAT1A in *Chlamydomonas*

The coding sequence of *NoDGAT1A* was amplified and cloned into *Nde*I/*Eco*RI sites of pOpt_Clover_Hyg [73], followed by sequencing for verification. The resulting plasmid was linearized by *Xba*I and transformed into the *Chlamydomonas* strain UVM4 [42] via the glass beads method [74]. Transformants were selected on Tris–acetate–phosphate (TAP) plates with 10 μg mL^−1^ hygromycin B (Sigma-Aldrich). The integration of *NoDGAT1A* into *Chlamydomonas* genome was verified by genomic PCR, and its expression was determined by quantitative real-time PCR. To impose nitrogen depletion, UVM4 cells in the stationary growth phase were collected and washed with nitrogen-free TAP medium (TAP-N), resuspended in fresh TAP-N, and cultured at 23 °C under continuous illumination of 40 µE m^−2^ s^−1^.

### Lipid extraction and analysis

Lipid extracts from yeast, *N. oceanica,* and *C. reinhardtii* cells as well as the in vitro DGAT assay mixture were all performed according to our previously described procedures [26, 30].

Neutral lipids were separated on a Silica gel 60 TLC plate (EMD Chemicals, Merck, Darmstadt, Germany) using a mixture of hexane/tert-butylmethyl ether/acetic acid (80/20/2, by volume) as the mobile phase, while polar lipids were separated on a TLC plate using a mixture of chloroform/methanol/acetic acid/water (25/4/0.7/0.3, by volume) as the mobile phase. Lipid detection by charring and lipid quantification by gas chromatography–mass spectrometry (GC–MS) were performed as described previously by Liu et al. [26]. TAG positional analysis was performed using *R. arrhizus* lipase (Sigma-Aldrich) following the procedures of Li et al. [58].

### Statistical analysis

All experiments were determined in biological triplicate to ensure the reproducibility. Experimental results were obtained as the mean value ± SD. Statistical analyses were performed using the SPSS statistical package (SPSS Inc., Chicago, IL, USA). Paired-samples *t*-tests were used for two group means, and one-way ANOVA Tukey’s HSD test was used for more than two group means. The statistical significances were achieved when P < 0.05.

## Additional files

**Additional file 1: Figure S1.** The ultrastructure of *N. oceanica* cells under nitrogen-replete (left) and nitrogen-depleted (right) conditions. **Figure S2.** GC-MS chromatogram of fatty acids in the *sn*-2 and *sn*-1/*sn*-3 positions of TAG from *N. oceanica* cells under nitrogen-replete (+N) and nitrogen-depleted (-N) conditions. **Figure S3.** Cladogram of the DGATs from plants, fungi, algae and animals. The neighbor-joining method was used to reconstruct the cladogram using MEGA6 [34], with the bootstrap value (obtained from 1000 replicates) is shown on each node. The scale bar 0.1 represents 10% divergence, calculated as the estimated number of replacement. Circles, plants; Squares, fungi; Filled circles, algae; triangles, animals. Protein sequences used for the cladogram construction see Additional file 2: Table S1. **Figure S4.** Predicated transmembrane domains for NoDGAT1A NoDGAT1B by TMHMM (V2.0, http://www.cbs.dtu.dk/services/TMHMM/). **Figure S5.** Protein sequence alignment of putative DGAT1s. The alignment was conducted using ClustalX2.1. The sequences used see Additional file 2: Table S1. Red arrows indicate the key amino acid residues identified by previous studies. **Figure S6.** The transcriptional expression levels of *NoDGAT1A and NoDGAT1B* in H1246 (upper) and INVSc1 (lower), as determined by quantitative real-time PCR. The gene expression levels were normalized to the endogenous *ACT1* gene. **Figure S7.** TAG content per dry weight (**a**) and the ratio of fatty acids in TAG over TFA (**b**) in *NoDGAT1A* knockdown lines and EV. Algal cells grown in nitrogen-replete medium for 4 days (considered as 0 h of nitrogen depletion) were used for nitrogen depletion experiment. Data are expressed as mean ± SD (*n*=3). Asterisks indicate the significant difference compared with EV (*t*-test, P<0.05). **Figure S8.** TAG content per dry weight (**a**) and the ratio of fatty acids in TAG over TFA (**b**) in *NoDGAT1A* overexpression lines and EV. Algal cells grown in nitrogen-replete medium for 4 days (considered as 0 h of nitrogen depletion) were used for nitrogen depletion experiment. Data are expressed as mean ± SD (*n*=3). Asterisks indicate the significant difference compared with EV (*t*-test, P<0.05). **Figure S9.** TAG content per dry weight (**a**) and the ratio of fatty acids in TAG over TFA (**b**) in *NoDGAT1A* heterologous expression lines of *Chlamydomonas* and EV. Algal cells grown in nitrogen-replete medium for 4 days (considered as 0 h of nitrogen depletion) were used for nitrogen depletion experiment. Data are expressed as mean ± SD (*n*=3). Asterisks indicate the significant difference compared with EV (*t*-test, P<0.05). **Figure S10.** Time course of total fatty acid (TFA) content of *N. oceanica* in response to nitrogen depletion. **Figure S11.** Effect of cerulenin on TAG content of *N. oceanica* in response to nitrogen depletion. The cerulenin concentration used was 10 μM. **Figure S12.** TLC analysis of lipids extracted from *NoDGAT1B*-carrying H1246 cells without feeding (WF) or fed with free fatty acids of C18:2, C18:3, C20:4, or C20:5 (125 µM). *NoDGAT1A*-carrying H1246 cells were used as the positive control. **Figure S13.** TLC analysis of lipids resulting from *in vitro* enzymatic reactions of NoDGAT1B with various acyl-CoAs. C18:1/C16:0-DAG was used as the acyl acceptor; NoDGAT1A was used as the positive control (C16:0 as the acyl donor). **Figure S14.** Fatty acid composition of TAG in *N. oceanica* upon nitrogen depletion. **Figure S15.** The contents of polar lipids in EV and NoDGAT1A-i19 under nitrogen-depleted conditions (24 h). **Figure S16.** Schematic illustration of constructs for subcellular localization (**a**), knockdown (**b**), and overexpression (**c**) of NoDGAT1A in *N. ocenica* cells.

**Additional file 2: Table S1.** DGAT protein sequences used for the construction of phylogenetic tree in additional file 1: Figure S3. **Table S2.** Primers used in the present study. Underlined sequences designate the restriction enzyme sites. The sequences in box indicate the linker fragment introduced before GFP coding sequence.

## Abbreviations

cER: chloroplast endoplasmic reticulum
CoA: coenzyme A
DAG: diacylglycerol
DGAT: diacylglycerol acyltransferase
DGAT1: type-I DGAT
DGAT2: type-II DGAT
DGAT3: type-III DGAT
DGDG: digalactosyl diacylglycerol
DGTS: diacylglycerol-N,N,N-trimethylhomoserine
ER: endoplasmic reticulum
EV: empty vector
GC-MS: gas chromatography–mass spectrometry
GFP: green fluorescent protein
LD: lipid droplet
MGDG: monogalactosyl diacylglycerol
PAF: plastid autofluorescence
PC: phosphatidylcholine
PCR: polymerase chain reaction
PE: phosphatidylethanolamine
PG: phosphatidylglycerol
PI: phosphatidylinositol
SQDG: sulfoquinovosyldiacylglycerol
TAG: triacylglycerol
TAP: tris–acetate–phosphate
TFA: total fatty acids
TLC: thin-layer chromatography
VCP2: violaxanthin/chlorophyll a-binding protein 2
