# Supplementary material for: A type-I diacylglycerol acyltransferase modulates triacylglycerol biosynthesis and fatty acid composition in the oleaginous microalga, Nannochloropsis oceanica
Source: Biotechnol Biofuels. 2017 Jul 5;10:174. doi: 10.1186/s13068-017-0858-1 (PMC5499063; doi:10.1186/s13068-017-0858-1)
Supplement: Supplementary file 1 — Additional file 1: Figure S1. The ultrastructure of N. oceanica cells under nitrogen-replete (left) and nitrogen-depleted (right) conditions. Figure S2. GC-MS chromatogram of fatty acids in the sn-2 and sn-1/sn-3 positions of TAG from N. oceanica cells under nitrogen-replete (+N) and nitrogen-depleted (-N) conditions. Figure S3. Cladogram of the DGATs from plants, fungi, algae and animals. The neighbor-joining method was used to reconstruct the cladogram using MEGA6 [34], with the bootstrap value (obtained from 1000 replicates) is shown on each node. The scale bar 0.1 represents 10% divergence, calculated as the estimated number of replacement. Circles, plants; Squares, fungi; Filled circles, algae; triangles, animals. Protein sequences used for the cladogram construction see Additional file 2: Table S1. Figure S4. Predicated transmembrane domains for NoDGAT1A NoDGAT1B by TMHMM (V2.0, http://www.cbs.dtu.dk/services/TMHMM/). Figure S5. Protein sequence alignment of putative DGAT1s. The alignment was conducted using ClustalX2.1. The sequences used see Additional file 2: Table S1. Red arrows indicate the key amino acid residues identified by previous studies. Figure S6. The transcriptional expression levels of NoDGAT1A and NoDGAT1B in H1246 (upper) and INVSc1 (lower), as determined by quantitative real-time PCR. The gene expression levels were normalized to the endogenous ACT1 gene. Figure S7. TAG content per dry weight (a) and the ratio of fatty acids in TAG over TFA (b) in NoDGAT1A knockdown lines and EV. Algal cells grown in nitrogen-replete medium for 4 days (considered as 0 h of nitrogen depletion) were used for nitrogen depletion experiment. Data are expressed as mean ± SD (n=3). Asterisks indicate the significant difference compared with EV (t-test, P<0.05). Figure S8. TAG content per dry weight (a) and the ratio of fatty acids in TAG over TFA (b) in NoDGAT1A overexpression lines and EV. Algal cells grown in nitrogen-replete medium for 4 days (considered as 0 h [file 13068_2017_858_MOESM1_ESM.pdf]

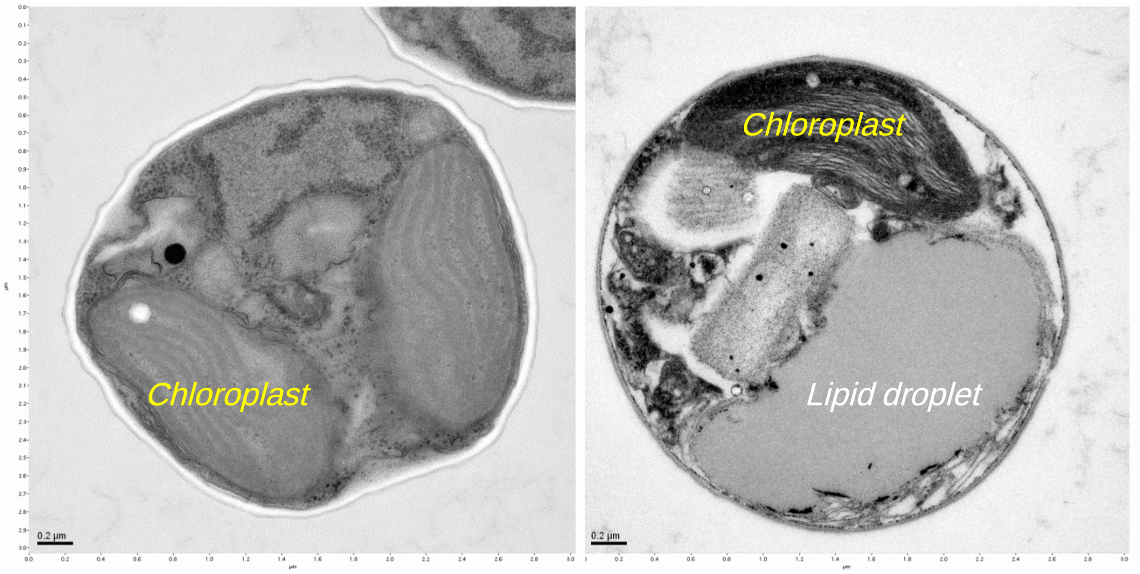

Figure S1. The ultrastructure of *N. oceanica* cells under nitrogen-replete (left) and nitrogen-depleted (right) conditions.

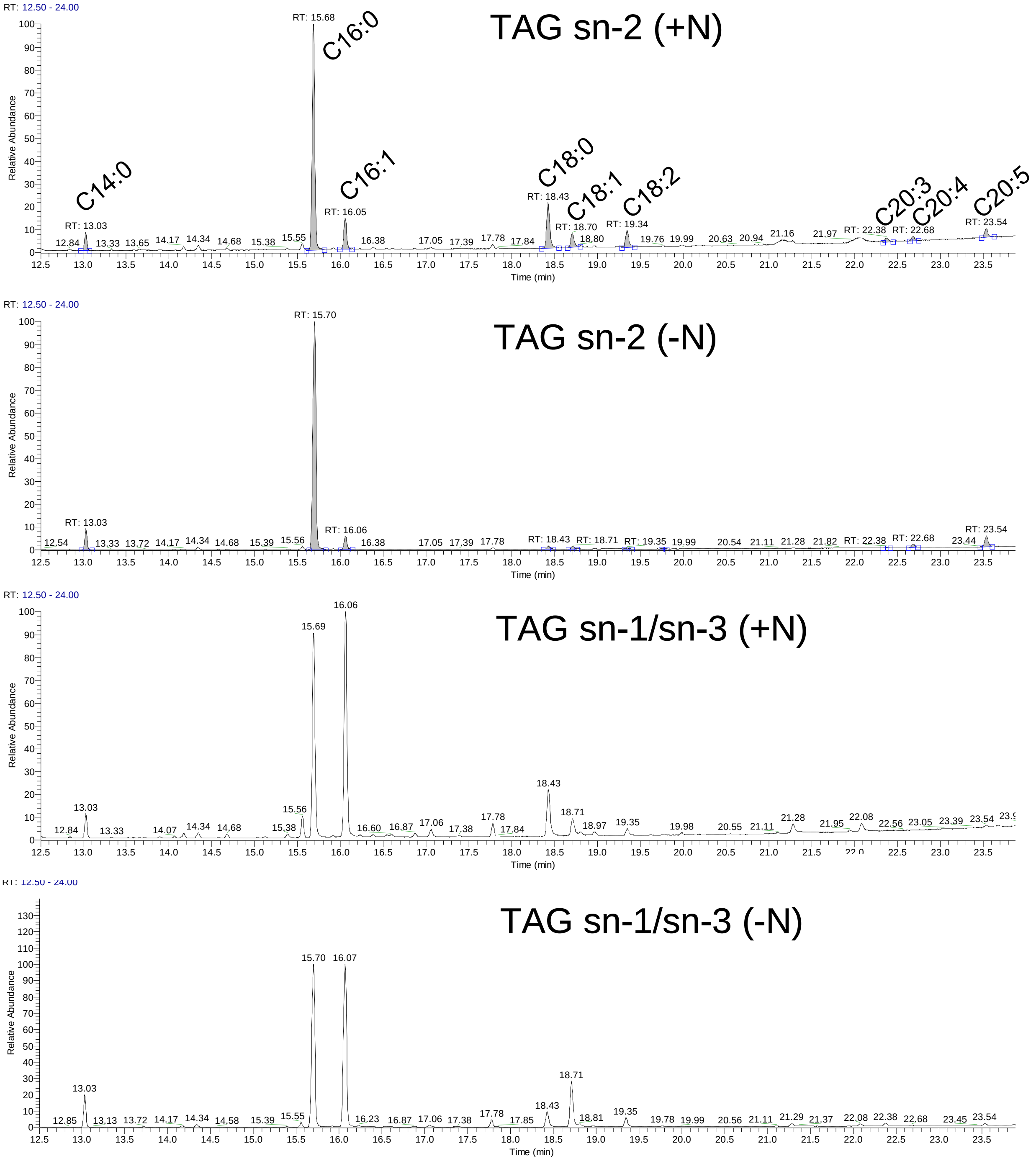

Figure S2. GC-MS chromatogram of fatty acids in the *sn*-2 and *sn*-1/*sn*-3 positions of TAG from *N. oceanica* cells under nitrate-replete (+N) and nitrate-depleted (-N) conditions

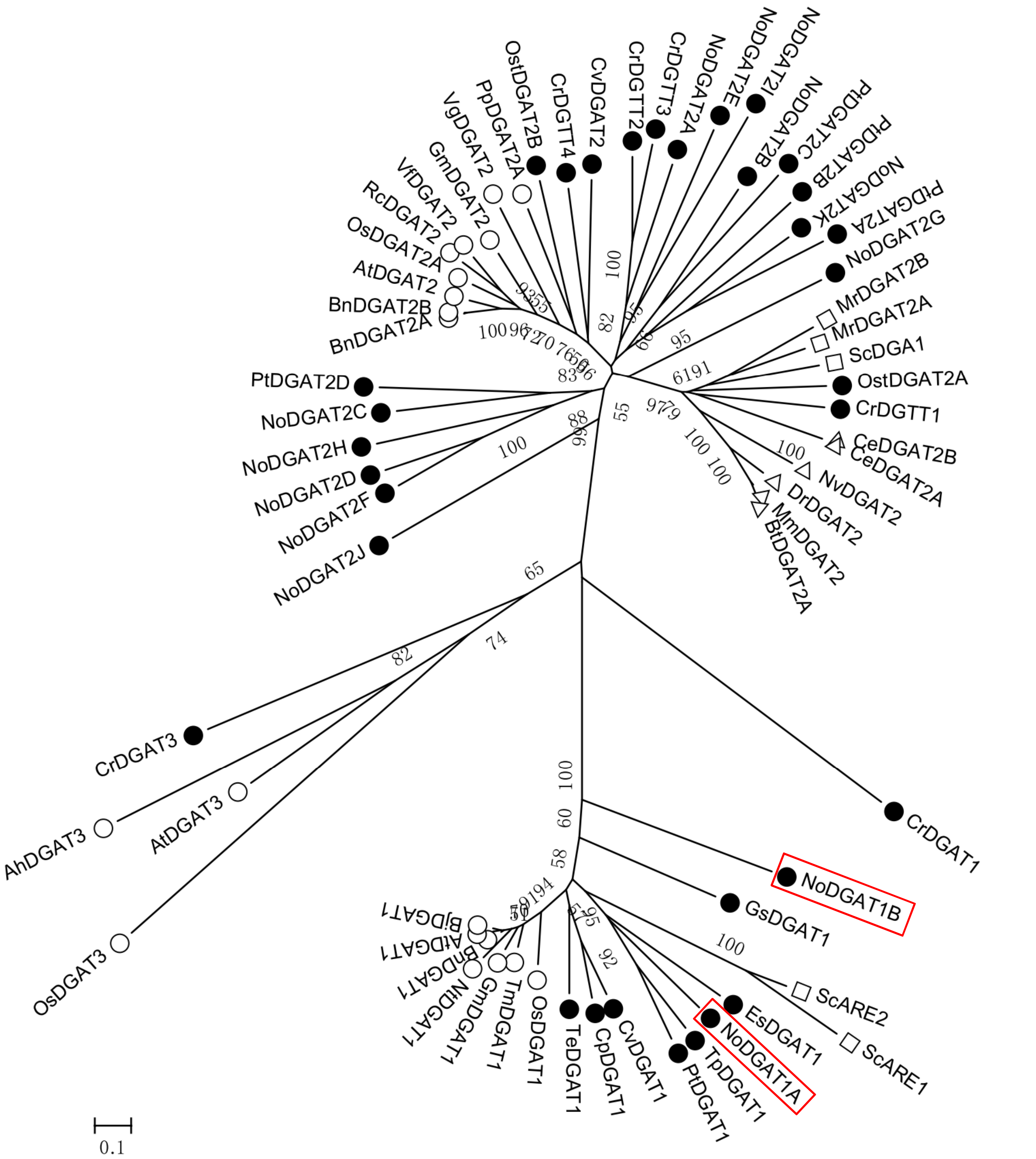

Figure S3. Cladogram of the DGATs from plants, fungi, algae and animals. The neighbor-joining method was used to reconstruct the cladogram using MEGA6 [34], with the bootstrap value (obtained from 1000 replicates) is shown on each node. The scale bar 0.1 represents 10% divergence, calculated as the estimated number of replacement. Circles, plants; Squares, fungi; Filled circles, algae; triangles, animals. Protein sequences used for the cladogram construction see Additional file 2: Table S1.

NoDGAT1A

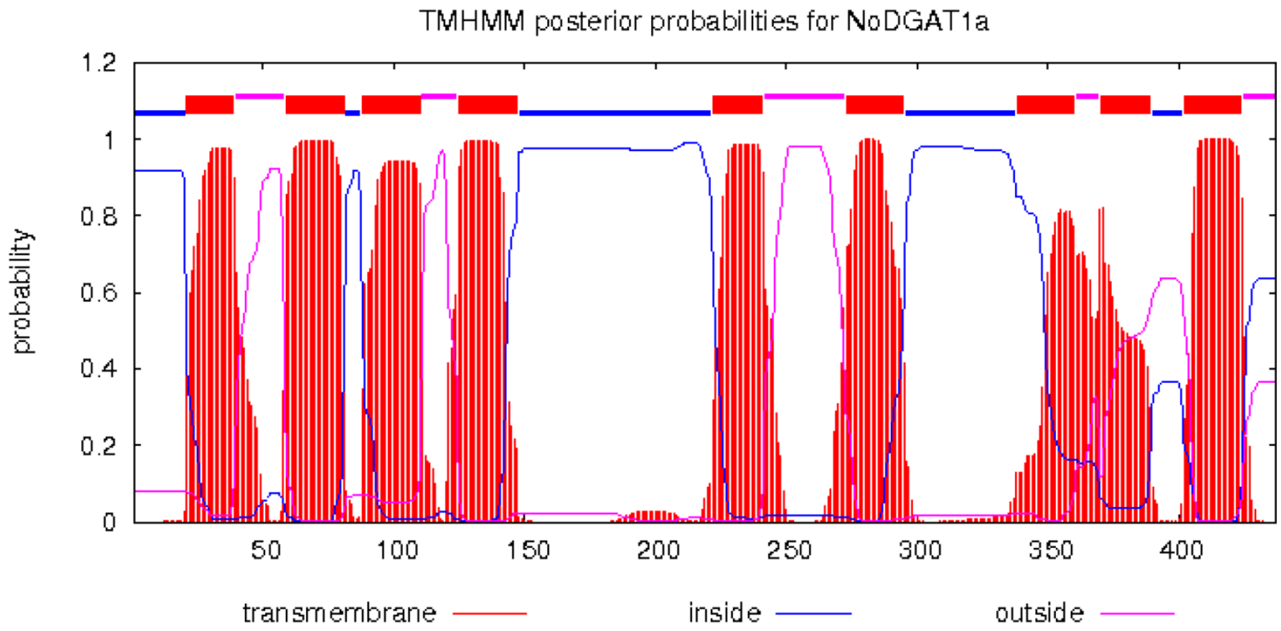

NoDGAT1B

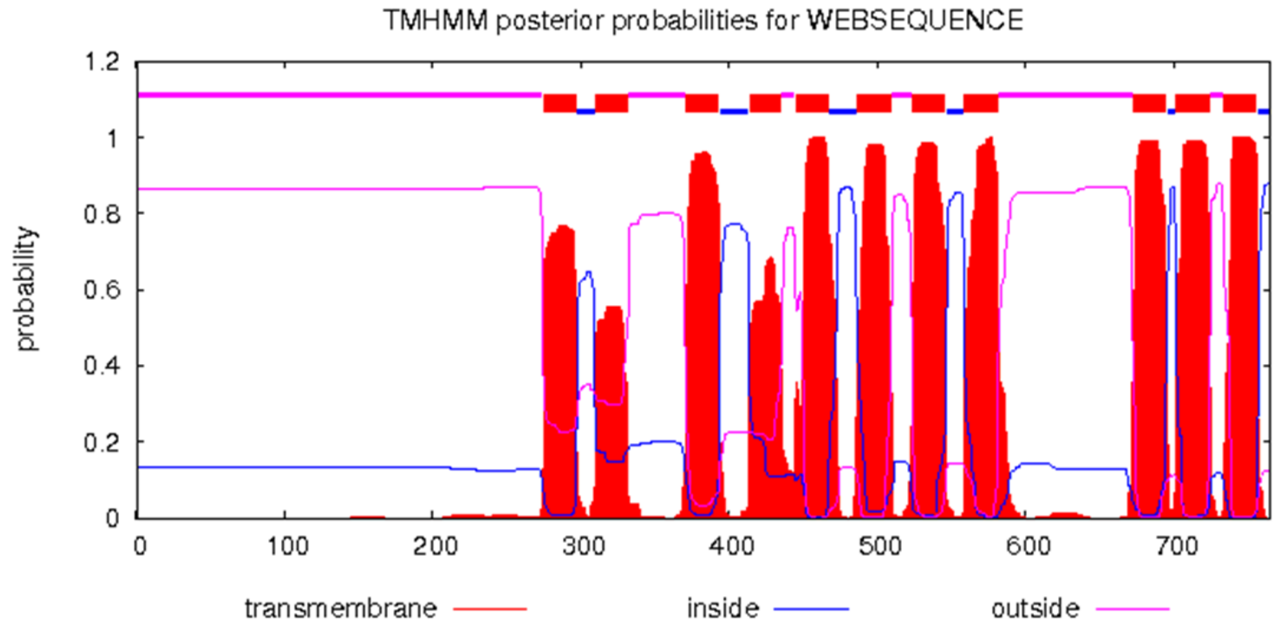

Figure S4 Predicated transmembrane domains for NoDGAT1A NoDGAT1B by TMHMM (V2.0, http://www.cbs.dtu.dk/services/TMHMM/).

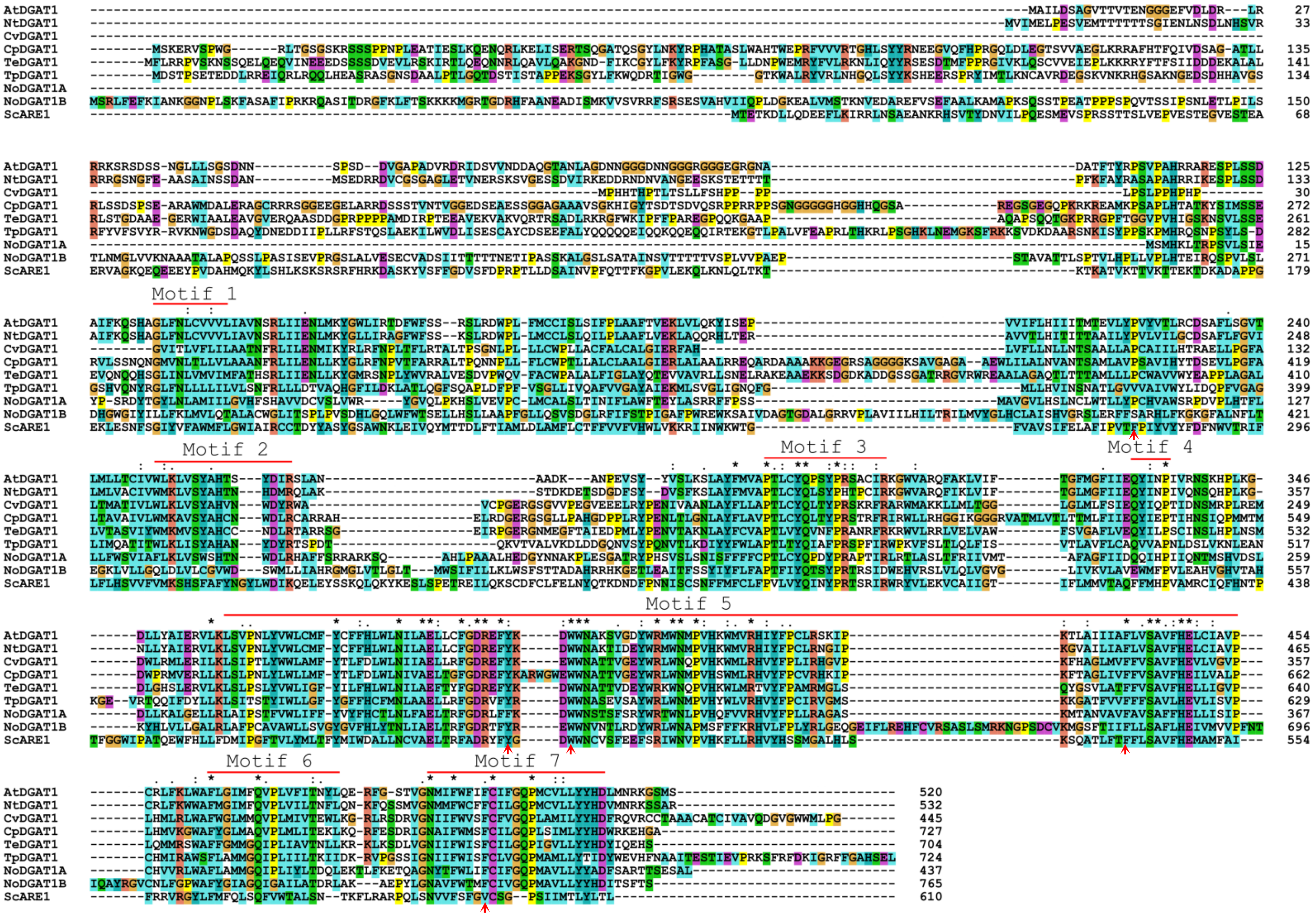

Figure S5 Protein sequence alignment of putative DGAT1s. The alignment was conducted using ClustalX2.1. The sequences used see Additional file 2: Table S1. Red arrows indicate the key amino acid residues identified by previous studies.

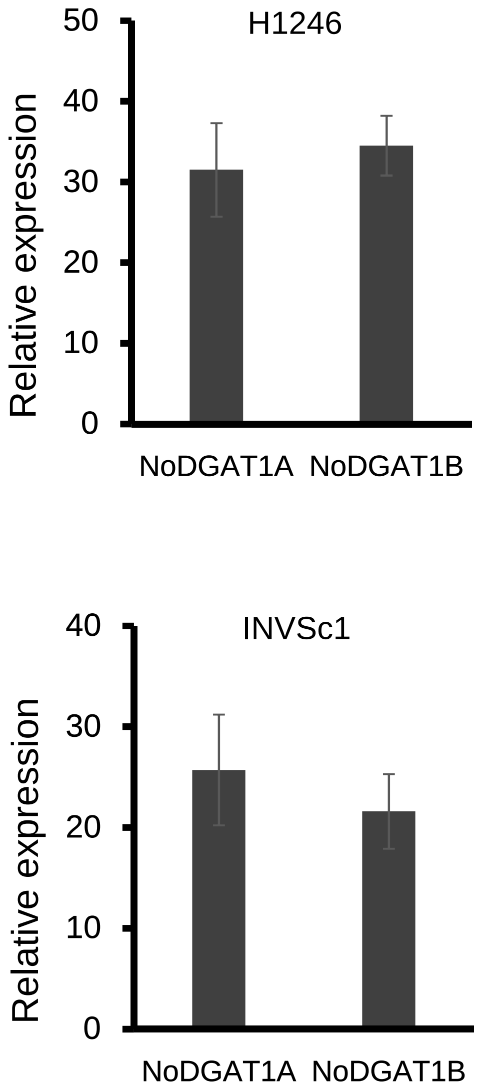

Figure S6. The transcriptional expression levels of *NoDGAT1A and NoDGAT1B* in H1246 (upper) and INVSc1 (lower), as determined by quantitative real-time PCR. The gene expression levels were normalized to the endogenous *ACT1* gene.

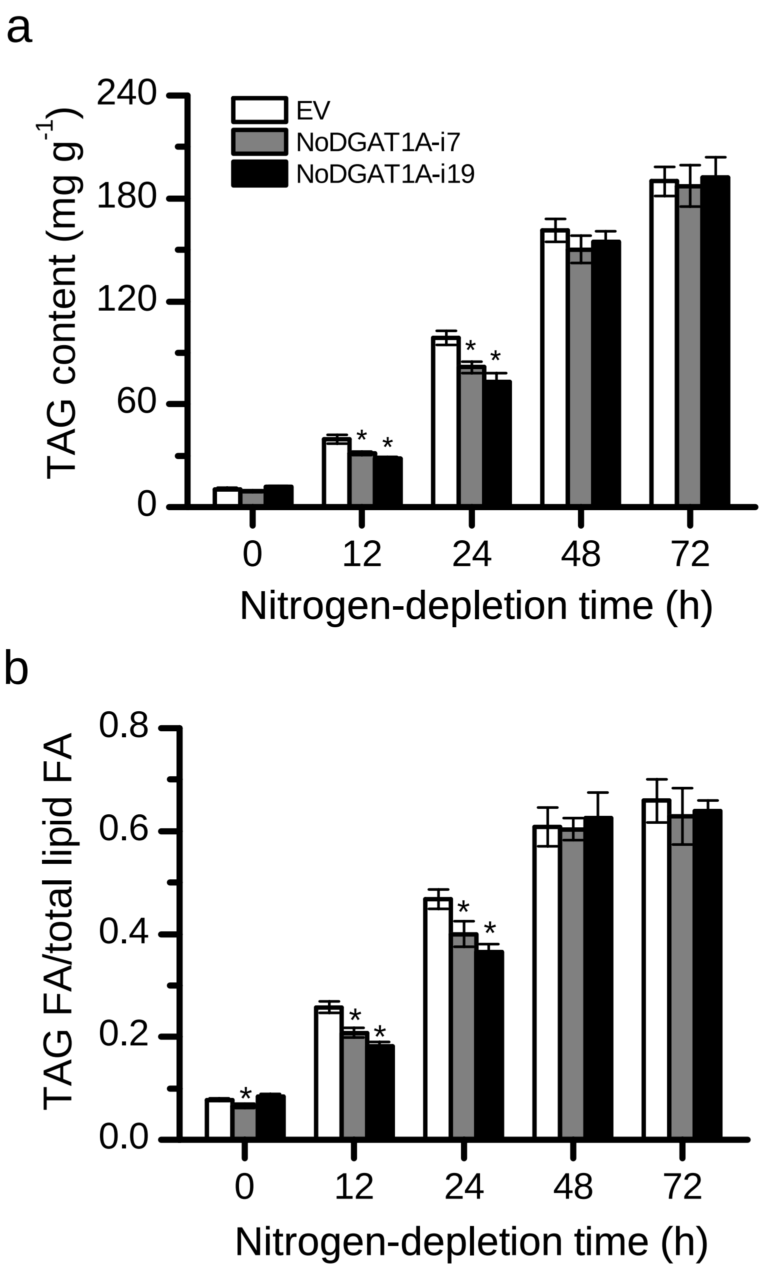

Figure S7. TAG content per dry weight (**a**) and the ratio of fatty acids in TAG over TFA (**b**) in *NoDGAT1A* knockdown lines and EV. Algal cells grown in nitrogen-replete medium for 4 days (considered as 0 h of nitrogen depletion) were used for nitrogen depletion experiment. Data are expressed as mean ± SD (n=3). Asterisks indicate the significant difference compared with EV (t-test, $P<0.05$).

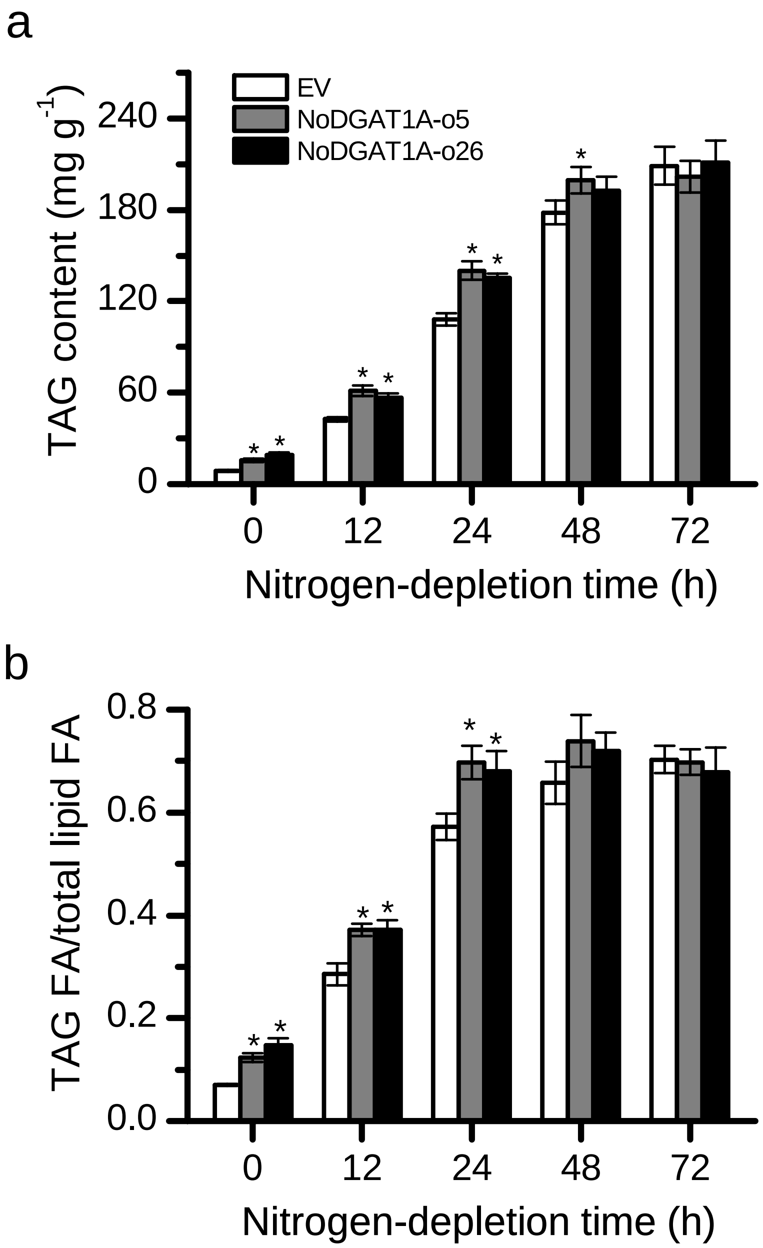

Figure S8. TAG content per dry weight (**a**) and the ratio of fatty acids in TAG over TFA (**b**) in *NoDGAT1A* overexpression lines and EV. Algal cells grown in nitrogen-replete medium for 4 days (considered as 0 h of nitrogen depletion) were used for nitrogen depletion experiment. Data are expressed as mean ± SD (*n*=3). Asterisks indicate the significant difference compared with EV (*t*-test, P<0.05).

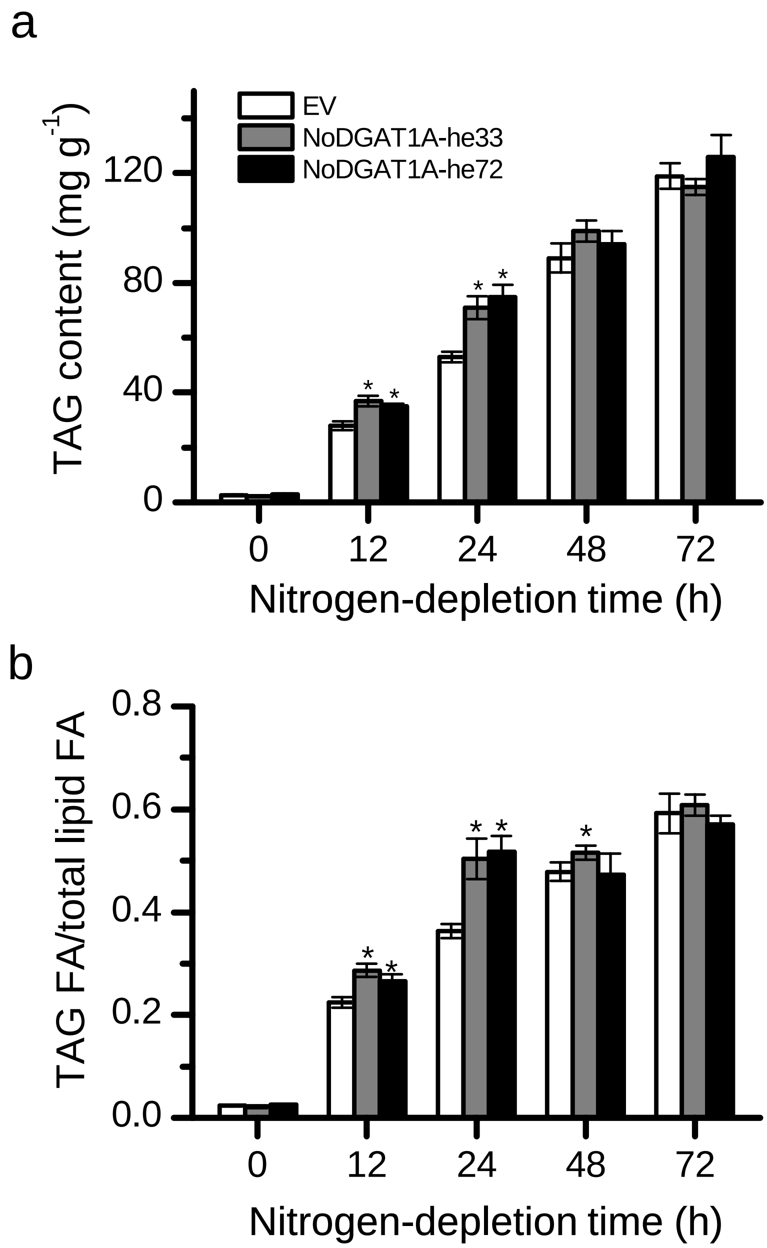

Figure S9. TAG content per dry weight (**a**) and the ratio of fatty acids in TAG over TFA (**b**) in *NoDGAT1A* heterologous expression lines of *Chlamydomonas* and EV. Algal cells grown in nitrogen-replete medium for 4 days (considered as 0 h of nitrogen depletion) were used for nitrogen depletion experiment. Data are expressed as mean ± SD (*n*=3). Asterisks indicate the significant difference compared with EV (*t*-test, P<0.05).

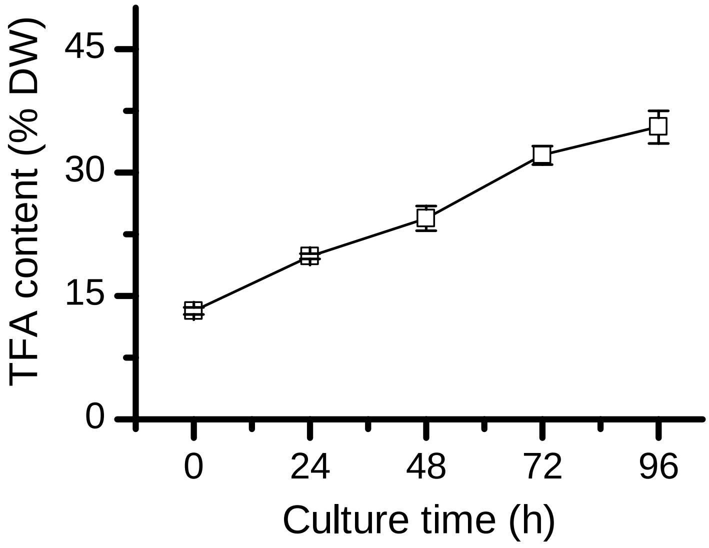

Figure S10. Time course of total fatty acid (TFA) content of *N. oceanica* in response to nitrogen depletion.

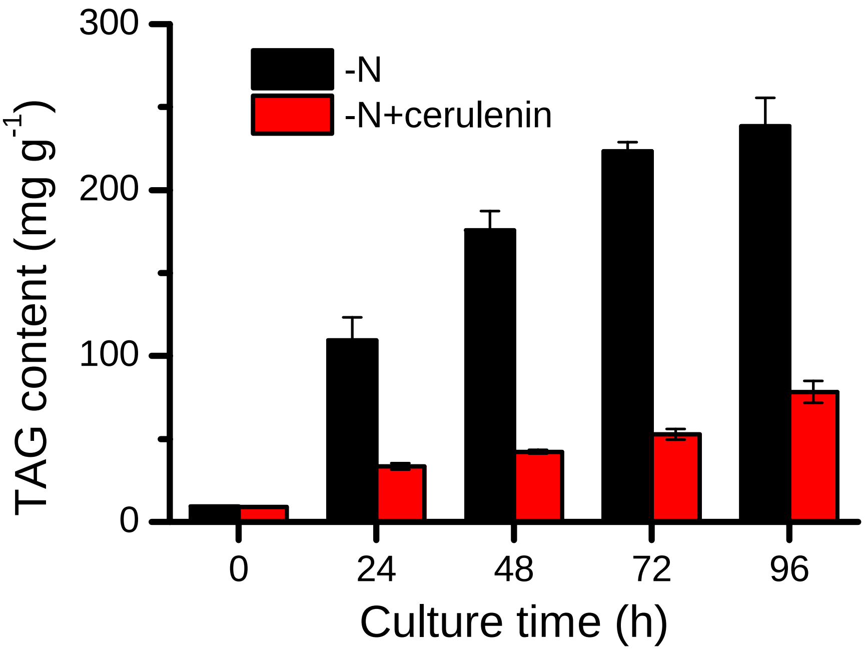

Figure S11. Effect of cerulenin on TAG content of *N. oceanica* in response to nitrogen depletion. The cerulenin concentration used was 10 μM.

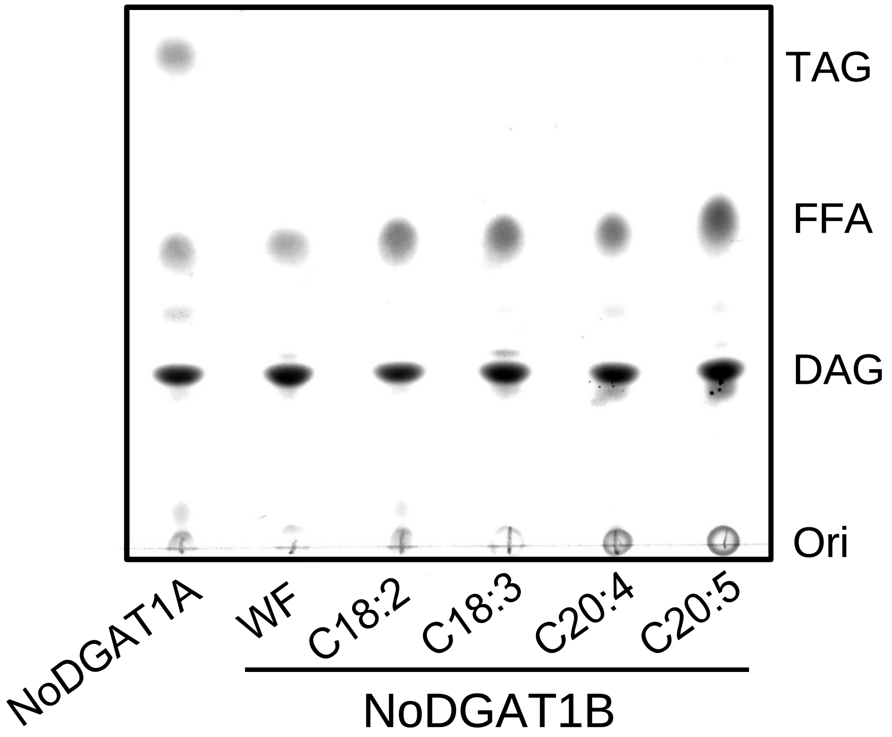

Figure S12. TLC analysis of lipids extracted from *NoDGAT1B*-carrying H1246 cells without feeding (WF) or fed with free fatty acids of C18:2, C18:3, C20:4, or C20:5 (125 µM). *NoDGAT1A*-carrying H1246 cells were used as the positive control.

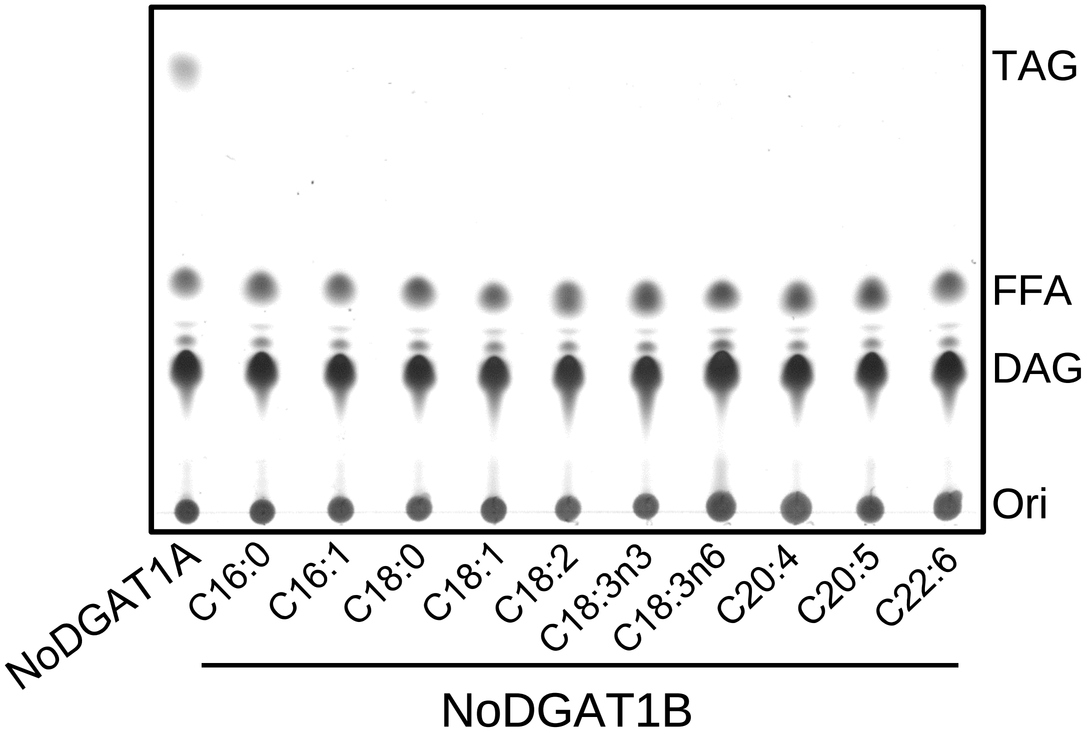

Figure S13. TLC analysis of lipids resulting from *in vitro* enzymatic reactions of NoDGAT1B with various acyl-CoAs. C18:1/C16:0-DAG was used as the acyl acceptor; NoDGAT1A was used as the positive control (C16:0 as the acyl donor).

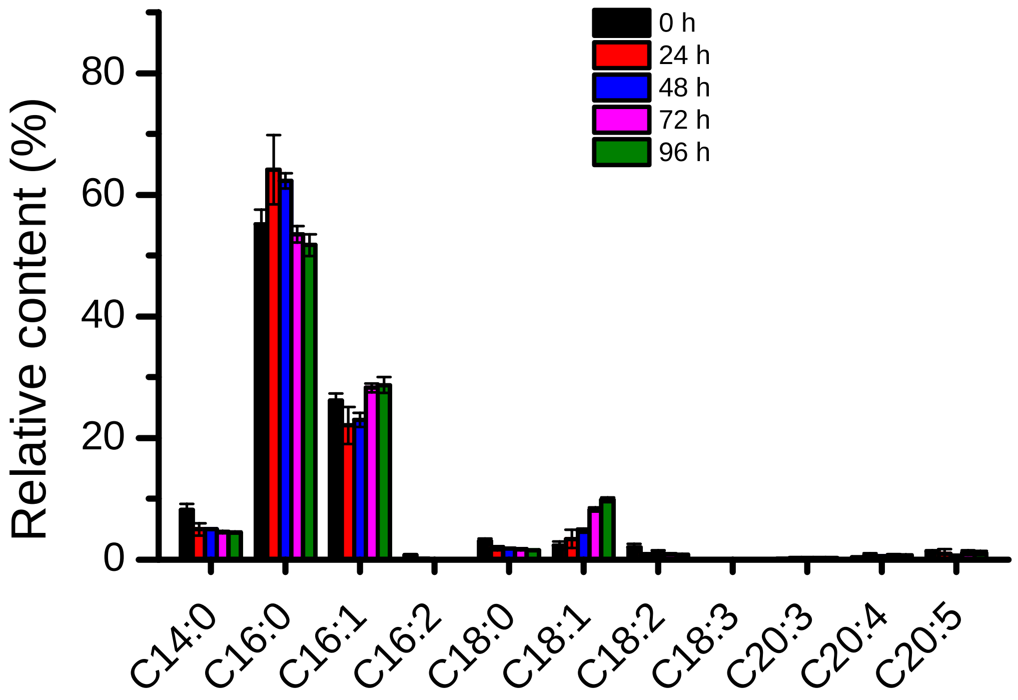

Figure S14. Fatty acid composition of TAG in *N. oceanica* upon nitrogen depletion

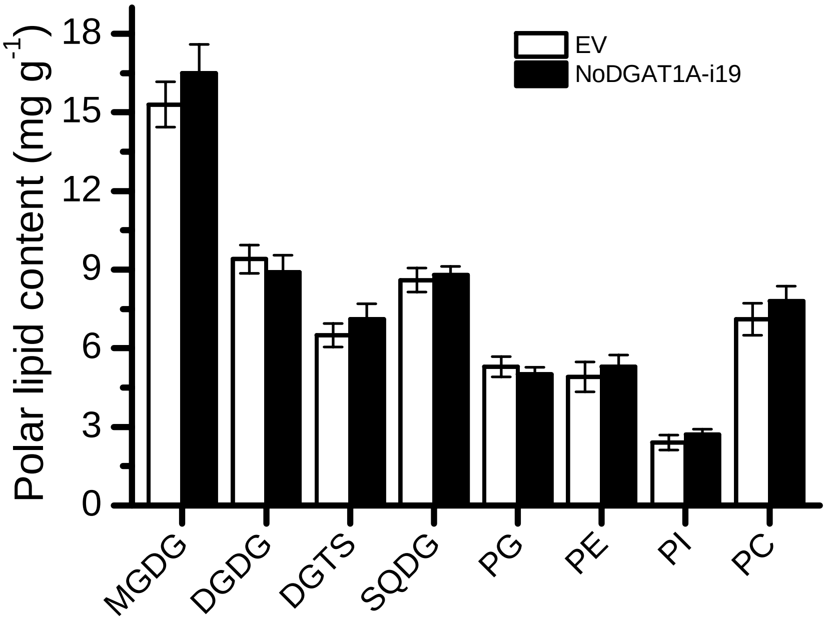

Figure S15 The contents of polar lipids in EV and NoDGAT1A-i19 under nitrogen-depleted conditions (24 h).

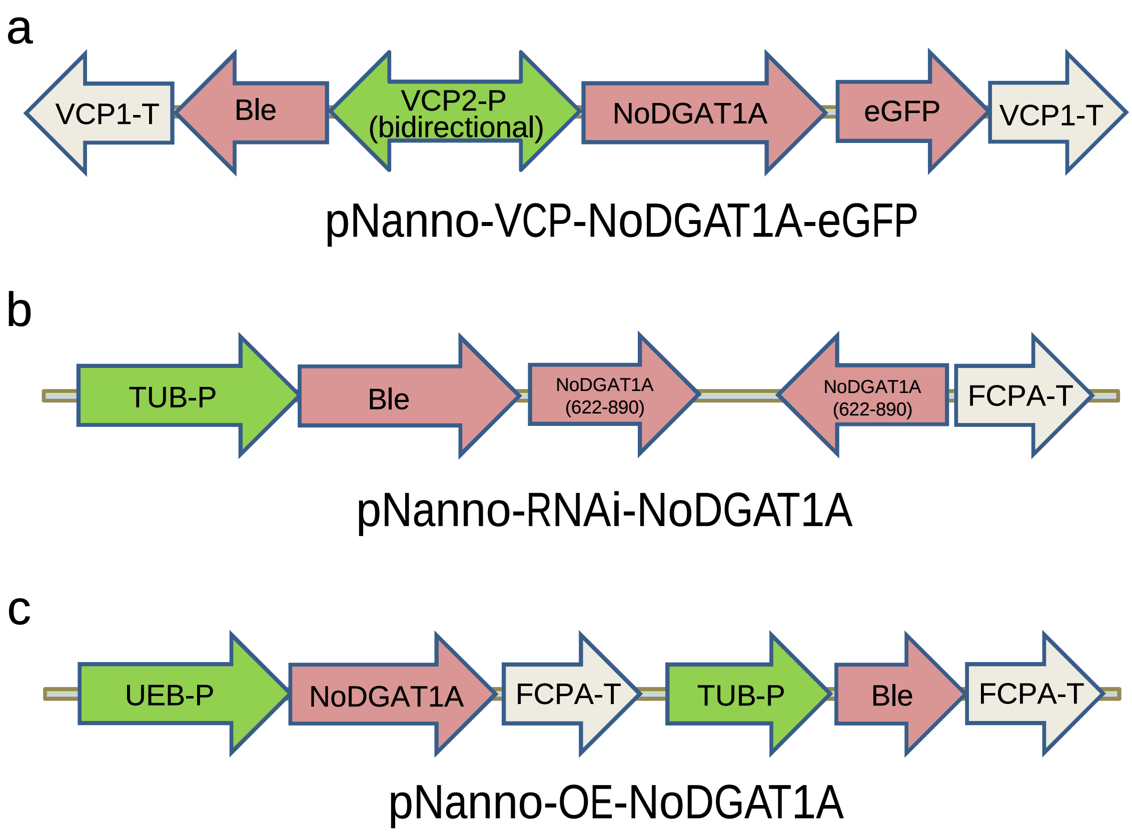

Figure S16 Schematic illustration of constructs for subcellular localization (**a**), knockdown (**b**), and overexpression (**c**) of NoDGAT1A in *N. oceanica* cells.
