# Supplementary material for: A type-I diacylglycerol acyltransferase modulates triacylglycerol biosynthesis and fatty acid composition in the oleaginous microalga, Nannochloropsis oceanica
Source: Biotechnol Biofuels. 2017 Jul 5;10:174. doi: 10.1186/s13068-017-0858-1 (PMC5499063; doi:10.1186/s13068-017-0858-1)
Supplement: Supplementary file 2 — Additional file 2: Table S1. DGAT protein sequences used for the construction of phylogenetic tree in additional file 1: Figure S3. Table S2. Primers used in the present study. Underlined sequences designate the restriction enzyme sites. The sequences in box indicate the linker fragment introduced before GFP coding sequence. [file 13068_2017_858_MOESM2_ESM.docx]

**Table S1** DGAT protein sequences used for the construction of phylogenetic tree in Additional file 1: Figure S3.

| Protein name | Source and Genbank Accession Number |
| --- | --- |
| AtDGAT1 | *Arabidopsis thaliana*, AAF19262 |
| BnDGAT1 | *Brassica napus*, AAF64065 |
| BjDGAT1 | *Brassica juncea*, AAY40784 |
| TmDGAT1 | *Tropaeolum majus*, AAM03340 |
| OsDGAT1 | *Oryza sativa*, AAW47581 |
| GmDGAT1 | Glycine max, AAS78662 |
| NtDGAT1 | *Nicotiana tabacum*, AAF19345 |
| ScARE1, 2 | *Saccharomyces cerevisiae, NP_009978, NP_014416* |
| CrDGAT1 | *Chlamydomonas reinhardtii*, Cre01.g045900 |
| TeDGAT1 | *Tetraselmis* sp, JAC66181 |
| CvDGAT1 | *Chlorella variabilis*, XP_005842809 |
| CpDGAT1 | *Chlorella protothecoides*, KFM28983 |
| PtDGAT1 | *Phaeodactylum tricornutum*, XP_002177753 (partial) |
| TpDGAT1 | *Thalassiosira pseudonana*, ADV58933 |
| EsDGAT1 | *Ectocarpus siliculosus*, CBN77837 (partial) |
| GsDGAT1 | *Galdieria sulphuraria,* XP_005706068 |
| AtDGAT2 | *Arabidopsis thaliana, NP_566952* |
| BnDGAT2A, 2B | *Brassica napus*, ACO90187, ACO90188 |
| GmDGAT2 | *Glycine max*, ACU20344 |
| OsDGAT2A | *Oryza sativa*, NP_001047917 |
| PpDGAT2A | *Physcomitrella patens*, XP_001758758 |
| RcDGAT2 | *Ricinus communis*, AAY16324 |
| VfDGAT2 | *Vernicia fordii*, ABC94473 |
| VgDGAT2 | *Vernonia galamensis*, ACV40232 |
| BtDGAT2A | *Bos taurus*, DAA21853 |
| MmDGAT2 | *Mus musculus*, NP_080660 |
| DrDGAT2 | *Danio rerio*, NP_001025367 |
| CeDGAT2A, 2B | *Caenorhabditis elegans*, NP_505413, NP_872180 |
| NvDGAT2C | *Nematostella vectensis*, XP_001635548 |
| ScDGA1 | *Saccharomyces cerevisiae*, NP_014888 |
| MrDGAT2A, 2B | *Mortierella ramanniana*, AAK84179, AAK84180 |
| CrDGTT1-4 | *Chlamydomonas reinhardtii*, Cre12.g557750, Cre02.g121200, Cre06.g299050, Cre03.g205050 |
| CvDGAT2 | *Chlorella variabilis*, EFN51306 |
| NoDGAT2A-2K | *Nannochloropsis oceanica*, http://www.bioenergychina.org/ |
| PtDGAT2A-2D | *P.tricornutum*, AFQ23659, AFM37314, AFQ23660, AFQ23661 |
| OstDGAT2A, 2B | *Ostreococcus tauri*, CAL56438, CAL58088 |
| AtDGAT3 | *A. thaliana*, AAK06873 |
| AhDGAT3 | *Arachis hipogaea*, AAX62735 |
| OsDGAT3 | *O. sativa*, AAS98422 |
| CrDGAT3 | *Chlamydomonas reinhardtii*, Cre06.g310200 |

**Table S2** Primers used in the present study.

| **Primer name** | **Sequences (5' to 3')** | **Purpose** |
| --- | --- | --- |
| NoDGAT1A_f1 | CAGCTCCCGAAGCATTCTCT | qPCR for NoDGAT1A |
| NoDGAT1A_r1 | CAGGGAATGCAGCACACCTA |  |
| NoDGAT1B_f1 | GCCAATGAAGCGGACATCTC | qPCR for NoDGAT1B |
| NoDGAT1B_r1 | CCTCCACGTTCTTGGTTGACA |  |
| NoActin_f | CCCAGGGAATGACAGTGCTT | qPCR for NoActin |
| NoActin_r | GCTCCCGTCAAAATCACGTT |  |
| NoDGAT1A_f2 (*Bam*HI) | GGCggatccATGTCTATGCACAAACTGACTCGACC | Cloning NoDGAT1A into pYES2-CT |
| NoDGAT1A_r2 (*Xba*I) | GGCtctagaCTAAAGAGCGCTCTCGCTGGTAGT |  |
| NoDGAT1B_f2 (*Bam*HI) | GGCggatccTGTCCTCTCTTGATACCCCT | Cloning NoDGAT1B into pYES2-CT |
| NoDGAT1B_r2 (*Xba*I) | GGCtctagACATACAGAGTGATACACACACCT |  |
| Nanno-GFP_f1 (*Kpn*I) | GGggtaccGGACCTAGGGGAGGAGGAGGAGGAATGGTGAGCAAGGGCGAG | For the construct of NoDGAT1A-GFP fusion |
| Nanno-GFP_r1 (*Kpn*I) | GGggtaccGAGAGAGTGGTGGAGTTGA |  |
| NoDGAT1A_f3 (*Kpn*I) | GGggtaccATGTCTATGCACAAACTG |  |
| NoDGAT1A_r3 (*Kpn*I) | GGggtaccAAGAGCGCTCTCGCTGGTA |  |
| NoDGAT1A_f4 (*Eco*RI) | GCCgaattcTACCAGCCCGACTACCC | For RNAi vector of NoDGAT1A |
| NoDGAT1A_r4 (*Xba*I) | GCCtctagaCCGAGACAGCGAAAA |  |
| NoDGAT1A_r4' (*Xba*I) | GCCtctagaAATTCCGCGAAAAGATTGA |  |
| UEP_f1 (*Eco*RI) | GGCgaattcGCTGCTGCCCCGAC | Amplifying the ubiquitin extension protein promoter |
| UEP_f2 (*Sac*I) | GGCgagctcATCCTGCTGTATGATTTTGG |  |
| NoDGAT1A_f5 (*Nde*I) | GGCcatatgATGTCTATGCACAAACTGACTCGACC | Heterologous expression vector for *Chlamydomonas* |
| NoDGAT1A_r5 (*Eco*RI) | GGCgaattcCTAAAGAGCGCTCTCGCTGGTAGT |  |

Underlined sequences designate the restriction enzyme sites. The sequences in box indicate the linker fragment introduced before GFP coding sequence.
